# A Natural Autophagy Activator *Castanea crenata* Flower Alleviates Skeletal Muscle Ageing

**DOI:** 10.1002/jcsm.13710

**Published:** 2025-01-28

**Authors:** So‐Hyun Park, Pyeong Geun Choi, Hee‐Soo Kim, Eunyoung Lee, Da‐Hye Lee, Min Jung Kim, Daedong Kim, Hyo‐Deok Seo, Jeong‐Hoon Hahm, Tae‐Il Jeon, Yang‐Hoon Huh, Jiyun Ahn, Tae‐Youl Ha, Chang Hwa Jung

**Affiliations:** ^1^ Aging and Metabolism Research Group Korea Food Research Institute Wanju‐gun Jeollabuk‐do Republic of Korea; ^2^ Department of Food Biotechnology University of Science and Technology Wanju‐gun Jeollabuk‐do Republic of Korea; ^3^ Department of Biochemistry, Molecular Biology and Biophysics University of Minnesota Minneapolis Minnesota USA; ^4^ Personalized Diet Research Group Korea Food Research Institute Wanju‐gun Jeollabuk‐do Republic of Korea; ^5^ Department of Animal Science Chonnam National University Gwangju Republic of Korea; ^6^ Electron Microscopy Research Center Korea Basic Science Institute Ochang Chungbuk Republic of Korea

**Keywords:** AMPK, autophagy, *Castanea crenata* flower, protein acetylation, sarcophenia

## Abstract

**Background:**

Sarcopenia, characterized by a gradual decline in skeletal muscle mass and function with age, significantly impacts both quality of life and mortality. Autophagy plays a crucial role in maintaining muscle health. There is growing interest in leveraging autophagy to mitigate muscle ageing effects. The impact of natural autophagy activators on skeletal muscle ageing remains elusive. This study aims to identify natural autophagy activators and assess their effects on skeletal muscle ageing.

**Methods:**

To discover novel autophagy activators, we screened 493 natural products and identified *Castanea crenata* flower extract (CCFE) as a promising candidate. We investigated the effect of CCFE on cellular senescence in C2C12 cells induced by etoposide. In animal experiments, aged mice (18 months old) were fed a diet supplemented with 0.1% and 0.2% CCFE for 3 months. We assessed exercise capacity, mitochondrial function and autophagic flux to determine the impact of CCFE on skeletal muscle ageing. The components present in CCFE were analysed using LC‐MS/MS, and their functional properties were examined.

**Results:**

CCFE enhanced autophagic flux (LC3II 80% increase, *p* < 0.05) and reduced senescence‐associated β‐galactosidase activity (32.78% decrease, *p* < 0.001). In aged mice, a 3‐month supplementation with CCFE improved muscle weight (18% increase, *p* < 0.05) and function (treadmill performance increased by 60%, *p* < 0.5; grip strength increased by 25%, *p* < 0.05). It alleviated mitochondrial dysfunction (basal oxygen consumption rate increased by 59%, *p* < 0.05) and restored autophagy. CCFE enhanced autophagy by activating AMPK (80% increase, *p* < 0.01) and inhibiting Atg5 protein acetylation (65% decrease, *p* < 0.001), with contributions from ellagic acid and polyamines. CCFE supplementation restored polyamine levels (serum spermidine increased from 0.98 ± 0.08 to 2.22 ± 0.05 μg/mL, *p* < 0.001) and increased urolithin levels (serum urolithin A increased from 0 to 18.79 ± 0.062 ng/mL, *p* < 0.001), metabolites produced by the gut microbiome from ellagic acid in aged mice.

**Conclusions:**

CCFE effectively suppressed skeletal muscle ageing by preventing mitochondrial dysfunction and restoring autophagic flux in aged mice. It achieved this by modulating AMPK and EP300 acetyltransferase activity, with contributions from its constituents, ellagic acid and polyamines. These findings highlight the potential of CCFE as a therapeutic agent for extending healthspan and mitigating sarcopenia, providing a basis for future clinical trials.

## 1 Introduction

Ageing leads to a progressive decline in physiological functions, leading to the accumulation of deleterious changes in organisms due to various stresses [1]. Skeletal muscle, which comprises approximately 40% of total body weight, decreases in mass and function with age, a phenomenon known as sarcopenia [2]. Sarcopenia increases the risk of mortality and falls, significantly diminishing the quality of life for the elderly [3, 4]. It is also associated with other age‐related conditions, such as diabetes and cardiovascular diseases [5, 6]. Therefore, preserving skeletal muscle health is crucial for extending healthspan, considering the ageing global population [7].

Macroautophagy/autophagy, a highly conserved catabolic process, plays an important role in maintaining intracellular homeostasis. When induced by starvation or other stressors, autophagy sequesters cargo within a double membrane, which is then degraded via lysosomal fusion [8]. Autophagy is regulated through translational and post‐translational modifications, with mechanistic target of rapamycin (MTOR) and adenosine monophosphate AMP‐activated protein kinase (AMPK) being key regulators of autophagy‐related protein phosphorylation [9]. Other post‐translational modifications, such as ubiquitination, acetylation and glycosylation, are also emerging as important autophagy regulators [10]. Autophagy's role in ageing has garnered significant attention. For instance, it is implicated in dietary restriction‐induced lifespan extension in *Caenorhabditis elegans* , and rapamycin (RM), a well‐known autophagy inducer, has been shown to extend lifespan [11, 12]. In skeletal muscles, autophagy is crucial for maintaining stemness in muscle stem cells during ageing and for the selective degradation of mitochondria through mitophagy, which is important for maintaining skeletal muscle function and mitochondrial quality [13, 14].

Recent studies have suggested that natural products and their constituent compounds suppress ageing by activating autophagy. These products contain diverse bioactive compounds and offer the advantages of low side effects and high efficiency owing to synergistic effects [15]. For instance, purple sweet potato and grape skin extracts extended the lifespan of *Drosophila melanogaster* models and those with Parkinson's disease by activating autophagy and mitophagy [16, 17]. Tomatidine has extended the lifespan and healthspan of *C. elegans* by inducing mitophagy, and resveratrol has inhibited H_2_O_2_‐induced cellular senescence through autophagy [18, 19]. However, the effects of autophagy‐activating natural products on sarcopenia and their underlying mechanisms remain unexplored. Therefore, this study aims to identify a novel autophagy‐activating natural product and analyse its effects and mechanisms on ageing.

## 2 Methods

### 2.1 Preparation of CCFE

*Castanea crenata* flowers were collected from Gongju‐si, Chungcheongnam‐do, Republic of Korea. A voucher specimen was deposited and registered at the Korea Food Research Institute under registration number KFRI‐MAT‐0054. The dried flowers were extracted at 80°C for 2 h using ten volumes of 50% ethanol, then concentrated using a rotary evaporator. The extracts were lyophilized and stored at −20°C until use.

### 2.2 Cell Culture and the Induction of Cellular Senescence

C2C12, HeLa and 293T cells were cultured in high glucose DMEM (Hyclone, SH30243.01) supplemented with 10% foetal bovine serum and penicillin–streptomycin and incubated at 37°C with 5% CO_2_. To investigate the effect of CCFE on etoposide‐induced cellular senescence, cells were treated with CCFE (50, 100 μg/mL) for 2 before adding 0.5 μM of etoposide and incubating for 24 h. Cells were then washed with PBS thrice and incubated for 48 h in fresh medium. Senescence was assessed using a senescence‐associated β‐galactosidase (SA β‐gal) staining kit (Cell Signalling Technology, 9860) following the manufacturer's instructions.

### 2.3 siRNA Transfection

Mouse *Atg5*‐targeting siRNA and non‐targeting siRNA were purchased from Dharmacon. C2C12 cells were transfected with siRNA using Lipofectamine RNAi Max (Thermo Fisher Scientific, 13778100) as per the manufacturer's protocol. After 24 h, the transfection medium was replaced with fresh medium, and cellular senescence was induced as described above.

### 2.4 Immunoblotting Analysis

Immunoblotting was performed as previously described  [S1]. Detailed protocol descriptions and information about the primary antibodies used are available in the supplementary methods in the Supporting information.

### 2.5 Quantitative Reverse Transcription Polymerase Chain Reaction

Total RNA was extracted using the RNeasy Mini Kit (QIAGEN, 74106) and the RNeasy Fibrous Tissue Mini Kit (QIAGEN, 74704), following the manufacturer's protocol. cDNA was synthesized using a ReverTra Ace® qPCR RT kit (Toyobo, TOFSQ‐20). Quantitative PCR was performed using the SYBR Green Real‐Time PCR Master Mix (Toyobo, FSQ‐101) on the ViiA 7 Real‐Time PCR System. The primers used are listed in Table S1. Relative RNA levels were calculated by normalizing to *18s* mRNA levels.

### 2.6 Lentiviral Preparation, Viral Infection and Stable Cell‐Line Generation

HeLa cells were stably transfected with the mCherry‐EGFP‐LC3B vector, as described previously [S2]. HeLa cells stably expressing mCherry‐EGFP‐LC3B were treated with CCFE for 2 h to evaluate autophagic flux. Fluorescence images were captured using a confocal microscope (Olympus, FV3000), and green and red dots were counted using the ImageJ software (NIH, Bethesda, Maryland, USA) with the spot colocalization ComDet plugin (version 0.5.5) [S3].

### 2.7 Animal Experiments

Male C57BL/6J mice, 2‐month‐old, were obtained from Orient Bio Co., Ltd. (Seongnam‐si, Korea). The mice were housed in a cage under constant conditions until they reached 18 months of age. They were then divided into three groups: aged control mice fed an AIN‐93M diet (21 M), aged mice fed an AIN‐93M diet with 0.1% CCFE (21 M + 0.1% CCFE), and aged mice fed an AIN‐93M diet with 0.2% CCFE (21 M + 0.2% CCFE). Additionally, young control mice matched to 5 months were also tested. To examine autophagic flux in vivo, the mice in each group were starved for 17 h and treated with leupeptin (30 mg/kg, Sigma, L2884) via intraperitoneal injection 4 h before sacrifice. The mice were anaesthetised using isoflurane and then euthanized. All animal experiments were approved by the Animal Care and Use Committee of the Korea Food Research Institute (KFRI‐M‐19030).

### 2.8 Treadmill and Grip Strength Tests

Treadmill tests were conducted using a rodent treadmill (Ugo Basile). Mice were acclimatized to the treadmill over 2 d by running at 5 m/min for 20 min each day without incline. On the third day, they started at 5 m/min; after 3 min, the speed was increased by 2 m/min every 3 min. Grip strength tests were conducted using a grip strength testing machine (GT3; BioSeb). Each mouse completed five trials, and the grip strength values were normalized to their body weights. The highest and lowest values were excluded from the calculation.

### 2.9 Immunofluorescence

Immunofluorescence staining of mouse skeletal muscle was performed as previously described  [S4], with slight modifications. For a comprehensive description of the protocol, refer to the supplementary methods in the Supporting Information.

### 2.10 Oxygen Consumption Rate in Muscle Fibre and Electron Flow Assay of Isolated Mitochondria

The OCR in isolated extensor digitorum longus (EDL) muscle and electron flow assays were conducted according to a previous study [S5, S6]. EDL muscles were digested with collagenase, and the isolated, digested, single muscle fibres were seeded into XF24 wells. OCR measurements were taken using a Seahorse XF24 analyser (Agilent), starting with basal OCR followed by sequential injections of 1‐μM oligomycin, 400‐nM FCCP, and 1‐μM rotenone. To assess the electron flow in skeletal muscle mitochondria, gastrocnemius‐derived mitochondria were prepared and exposed to XF24 well plates with specific substrates. To investigate the complex‐specific effects of CCFE, the mitochondria were sequentially treated with rotenone, succinate, antimycin A and ascorbate with 0.2‐mM TMPD, while OCR was continuously measured.

### 2.11 Transmission Electron Microscopy (TEM) Imaging

TEM imaging was performed as previously described [S2]. Complete details are available in the supplementary methods available in the Supporting information.

### 2.12 Histone Acetyltransferase EP300 Activity

Histone acetyltransferase EP300 activity was measured using an activity kit (ANASPEC, AS‐72172) following the manufacturer's instructions.

### 2.13 Immunoprecipitation

Protein G agarose beads (Thermo Scientific, 20399) were washed with PBS. The quantified lysate was then added to the beads and rotated for 30 min. After centrifugation at 4500 rpm for 30 s at 4°C, the lysate was transferred to a new tube. Freshly washed beads were mixed with acetyl‐lysine antibody and the pre‐cleared lysate, then rotated overnight at 4°C. Following another centrifugation at 4500 rpm for 30 s, the supernatant was discarded. The beads were washed, and the proteins bound to the beads were analysed by immunoblotting.

### 2.14 UPLC‐ESI‐MS/MS Analysis of Mouse Serum, Liver and Kidney

Mouse samples were acidified and prepared for LC‐MS/MS analysis. The prepared extracts (5 μL) were injected into a UPLC‐MS/MS system (Acquity UPLC, Waters Corp.) equipped with a BEH C18 column (2.1 × 100 mm, 1.7 μm, Waters) with a linear gradient of solvent A (1% [v/v] formic acid in water) and solvent B (1% (v/v) formic acid in acetonitrile), starting with 5% B for 1 min, increasing linearly to 30% B over 4 min, then to 90% B by 7 min, followed by re‐equilibration until 10 min. The column temperature was maintained at 40°C with a flow rate of 0.35 mL/min. MS settings were optimized using a cone voltage of 28 V, a capillary voltage of 3.0 kV and a collision energy of 21 V; the source temperature was set at 150°C, and the desolvation gas temperature was set at 500°C. Detailed descriptions of these protocol methods are provided in the supplementary methods available in the Supporting Information.

### 2.15 Statistical Analysis

Data are presented as the mean ± standard deviation for in vitro analyses or the mean ± standard error of the mean for in vivo analyses. Significant differences were assessed with Student's unpaired *t*‐test, and three or more independent groups were evaluated using one‐way analysis of variance with Tukey's post hoc multiple comparison test (*p* > 0.05), performed with GraphPad Prism 9 software (GraphPad Software, Inc.).

## 3 Results

### 3.1 CCFE Activates Autophagic Flux In Vitro

To identify novel autophagy activators, we treated Huh7 cells with 493 plant extracts from our house library (Table S2) in combination with chloroquine (CQ), which promotes the accumulation of autophagosomes, and then incubated for 24 h. Autophagy activity was assessed using a commercial autophagy detection kit, and fluorescence intensity was measured using a microplate reader. The data were normalized against CQ‐treated control cells (Figure S1A) or CQ + rapamycin‐treated cells (Figure S1B) and presented as a heatmap, with more intense red indicating a high fluorescence value, signifying increased autophagic activity. Several natural products demonstrating high autophagic activity were identified (Figure S1). To further validate the effects of these natural products on autophagic flux, we treated Huh7 cells with 24 selected natural products for 24 h and co‐treated them with CQ to examine the protein levels of LC3. Among the selected natural products, CCFE induced the greatest increase in LC3‐II protein levels, surpassing those observed with the positive control, RM (Figure S2). This finding underscores the potential of CCFE as a promising new autophagy activator, with potential applications in enhancing health and addressing skeletal muscle ageing.

**FIGURE 1:**
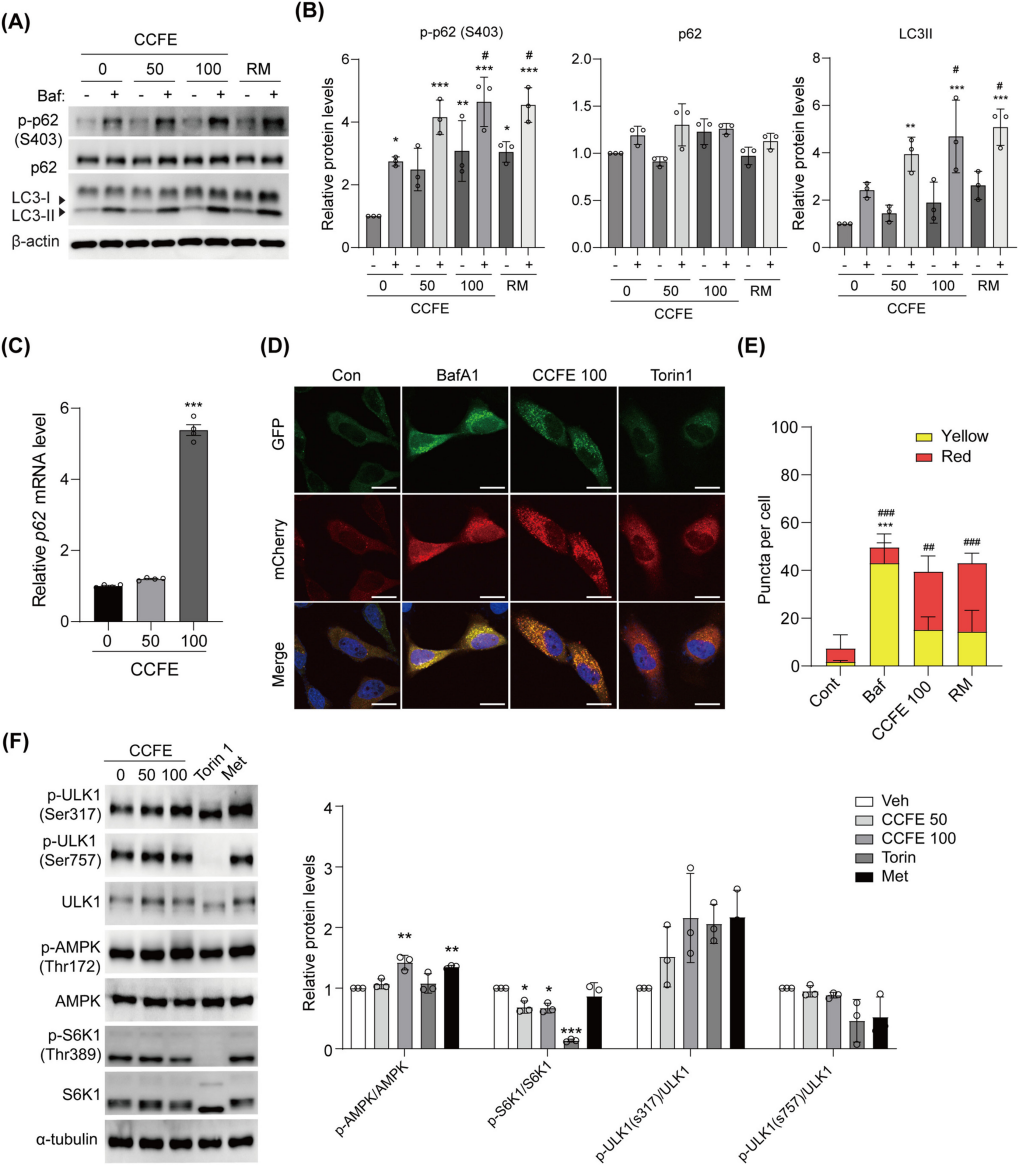
CCFE is a novel natural autophagy activator. (A) Protein expression of p‐p62, p62 and LC3 in C2C12 cells treated with CCFE (50–100 μg/mL) or rapamycin (RM, 50 nM) for 2 h in the presence or absence of bafilomycin A1 (Baf, 25 nM, 2 h).   (B) Quantified protein levels of p‐p62, p62 and LC3 from the western band (*n* = 3). *p < 0.05, **p < 0.01, ***p < 0.001 versus control, ^#^ *p* < 0.05 versus Baf‐treated control. (C) mRNA expression level of *p62*. (D) Cells expressing mCherry‐EGFP‐LC3 were treated with Baf (25 nM), CCFE (100 μg/mL) or Torin 1 (200 nM). After cell nucleus staining with DAPI, cells were examined for autophagic flux using confocal microscopy. (E) Number of red and yellow puncta examined in the merged images (*n* = 4). ***p < 0.001 versus control (yellow dot); ^#^ *p* < 0.05 versus control (red dot). (F) Protein expression of p‐ULK1 (S317), p‐ULK1 (S757), p‐AMPK (T172), AMPK, p‐S6K1, S6K1 in C2C12 cells treated with CCFE (50, 100 μg/mL), Torin 1 (200 nM) or metformin (Met, 500 μM) for 24 h and the quantification graph (n = 3). *p < 0.05, **p < 0.01, ***p < 0.001 versus control. Data are presented as mean ± SD.

The cytotoxicity of CCFE was evaluated in mouse myoblast C2C12 cells, revealing that concentrations below 200 μg/mL did not affect cell viability (Figure S3A). To investigate the effect of CCFE on autophagic flux, C2C12 cells were treated with CCFE (50–100 μg/mL) alongside RM (positive control). Additionally, cells were co‐treated with bafilomycin A1 (Baf), an autophagosome‐lysosome fusion inhibitor, for 2 h. CCFE treatment increased LC3II protein expression, and the Baf‐induced accumulation of autophagosomes was higher in CCFE‐treated cells than that in control cells (Figures 1A,B and S3B). While there was no significant difference in total p62 protein expression, phosphorylated p62 at serine 403 (p‐p62 [S403]) significantly increased in CCFE‐treated cells, both with and without Baf. p62 serves as a selective autophagy receptor protein that recognizes and transports ubiquitinated proteins to autophagosomes for degradation. Phosphorylation at serine 403 enhances p62 activity, and an increase in p‐p62 turnover indicates enhanced p62‐mediated autophagy flux [S7]. Enhanced autophagic flux typically reduces total p62 protein levels. To verify whether the increase in p62 protein levels in CCFE‐treated cells was ascribed to the inhibition of autophagic flux, we measured the mRNA levels of p62. The results showed a significant increase in p62 mRNA levels, suggesting that the rise in p62 protein was due to enhanced mRNA levels rather than a blocked autophagic flux (Figure 1C).

mCherry‐EGFP‐LC3B‐transfected HeLa cells were employed to assess whether CCFE could enhance autophagic flux without blocking autophagosome‐lysosome fusion. The stability of mCherry red fluorescent protein under acidic conditions ensures that only red fluorescence persists in autolysosomes [S8]. Confocal imaging revealed that, like Torin 1 (the positive control), CCFE increased the number of autolysosomes (red dots), whereas Baf induced the accumulation of autophagosomes (yellow dots) (Figure 1D). Quantification of LC3 dots confirmed a significant increase in autophagic flux due to CCFE (Figure 1E). The results were consistent with those observed in C2C12 cells transiently transfected with the mCherry‐GFP‐LC3 plasmid (Figure S3C,D). Further, the impact of CCFE on two key autophagy regulatory pathways, AMPK and MTOR, was explored. CCFE reduced the phosphorylation of S6K1, a downstream protein in the MTOR pathway and increased AMPK phosphorylation. CCFE also enhanced ULK1 phosphorylation at Ser317 by AMPK, suggesting that CCFE promotes autophagic flux by activating AMPK (Figure 1F). Collectively, these findings suggest that CCFE boosts autophagic flux primarily through AMPK activation.

### 3.2 CCFE Suppresses Cellular Senescence in Skeletal Muscle Cells

**FIGURE 2:**
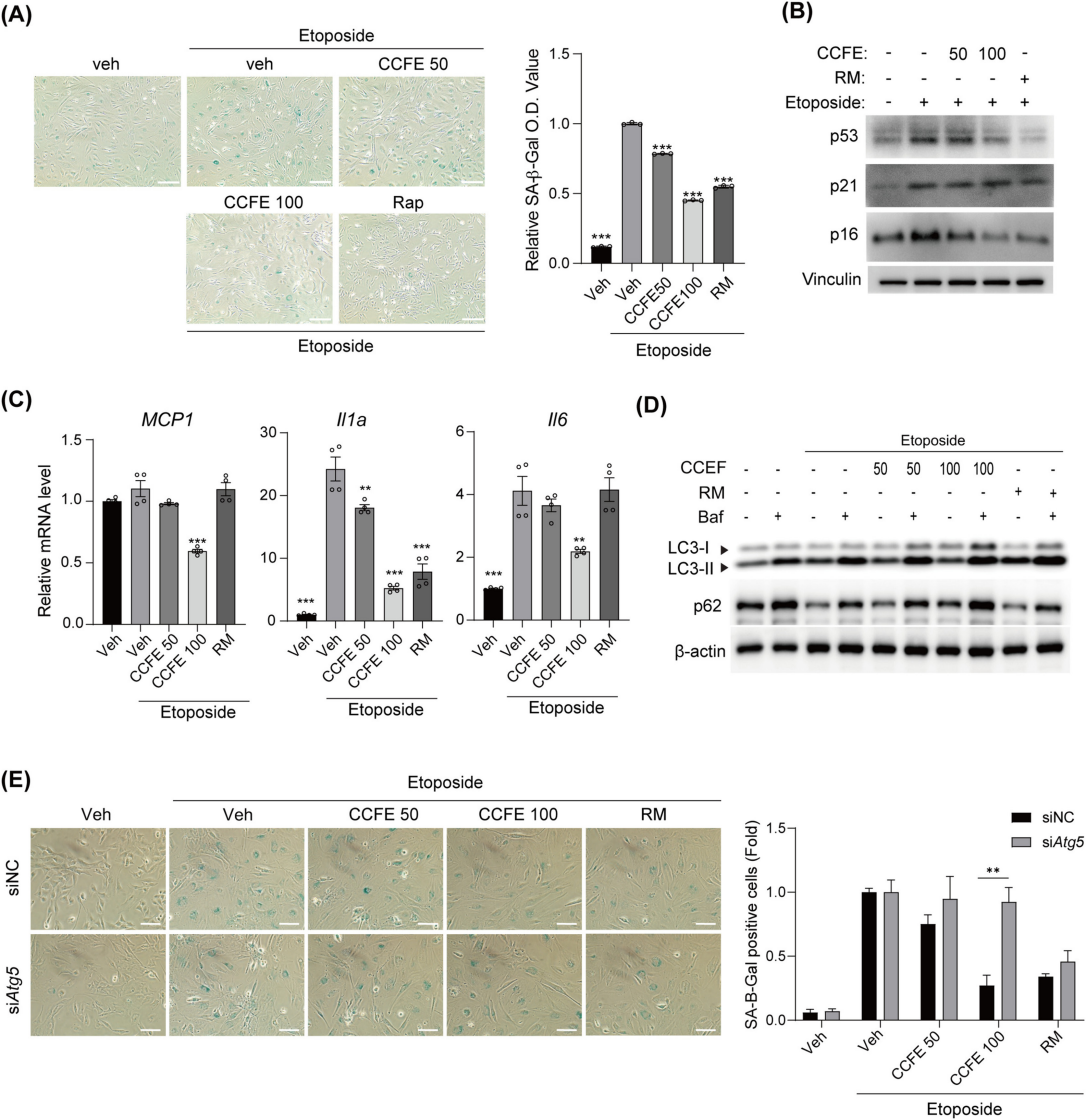
CCFE suppresses etoposide‐induced cellular senescence. (A) Senescence‐associated β‐galactosidase (SA β‐gal) staining image of normal and etoposide‐induced senescent cells treated with or without CCFE or RM (scale bar; 100 μm) and the relative OD value of SA β‐gal measured at 632 nm. (B) Protein expression of p53, p21 and p16; and (C) mRNA expression of *Mcp1*, *Il1a* and *Il6* in control and senescent cells treated with CCFE and RM. (D) LC3 and p62 protein expression levels in the presence or absence of Baf. (E) SA β‐gal staining images (scale bar; 100 μm) of etoposide‐induced senescent cells treated with CCFE or RM in siNC‐ and si*Atg5*‐transfected C2C12 cells and the quantified SA β‐gal positive cell (n = 3). Data are presented as mean ± SD. *p < 0.05, **p < 0.01, ***p < 0.001 versus etoposide‐induced senescent cell.

We investigated the potential inhibitory effects of CCFE on cellular senescence. To induce cellular senescence, we utilized etoposide, a topoisomerase II inhibitor known to cause DNA damage, and assessed the effects of CCFE on etoposide‐induced senescence in C2C12 cells using SA β‐gal staining [S9]. Etoposide effectively induced cellular senescence, whereas co‐treatment with CCFE reduced SA β‐gal activity (Figure 2A). Furthermore, CCFE lowered the cellular senescence‐related protein levels of p53 and p16 (Figure 2B) and reduced mRNA expression of *Mcp1*, *Il1a* and *Il6*, which are components of the senescence‐associated secretory phenotype (SASP), indicating CCFE's role in mitigating DNA damage‐induced senescence (Figure 2C). Additionally, we examined the effects of CCFE on senescent human skeletal muscle myoblasts induced by repeated passaging. Treatment with CCFE for 24 h led to reductions in both SA β‐gal activity and senescence‐associated protein levels (Figure S4A,B). We also assessed whether CCFE could enhance autophagic flux in these etoposide‐induced senescent cells. Analysis of LC3‐II expression levels with and without Baf treatment as part of an autophagy flux assay revealed that autophagic flux was impaired in etoposide‐treated cells but was restored by CCFE treatment (Figure 2D). To examine if CCFE inhibition of cellular senescence is mediated through autophagy, we investigated its effects in si*Atg5* cells, where autophagy was compromised. In these cells, the ability of CCFE to counteract etoposide‐induced cellular senescence was diminished (Figures 2E and S4C). Collectively, these findings suggest that CCFE suppresses cellular senescence and restores impaired autophagic flux in skeletal muscle cells.

### 3.3 CCFE Improved Skeletal Muscle Performance

**FIGURE 3:**
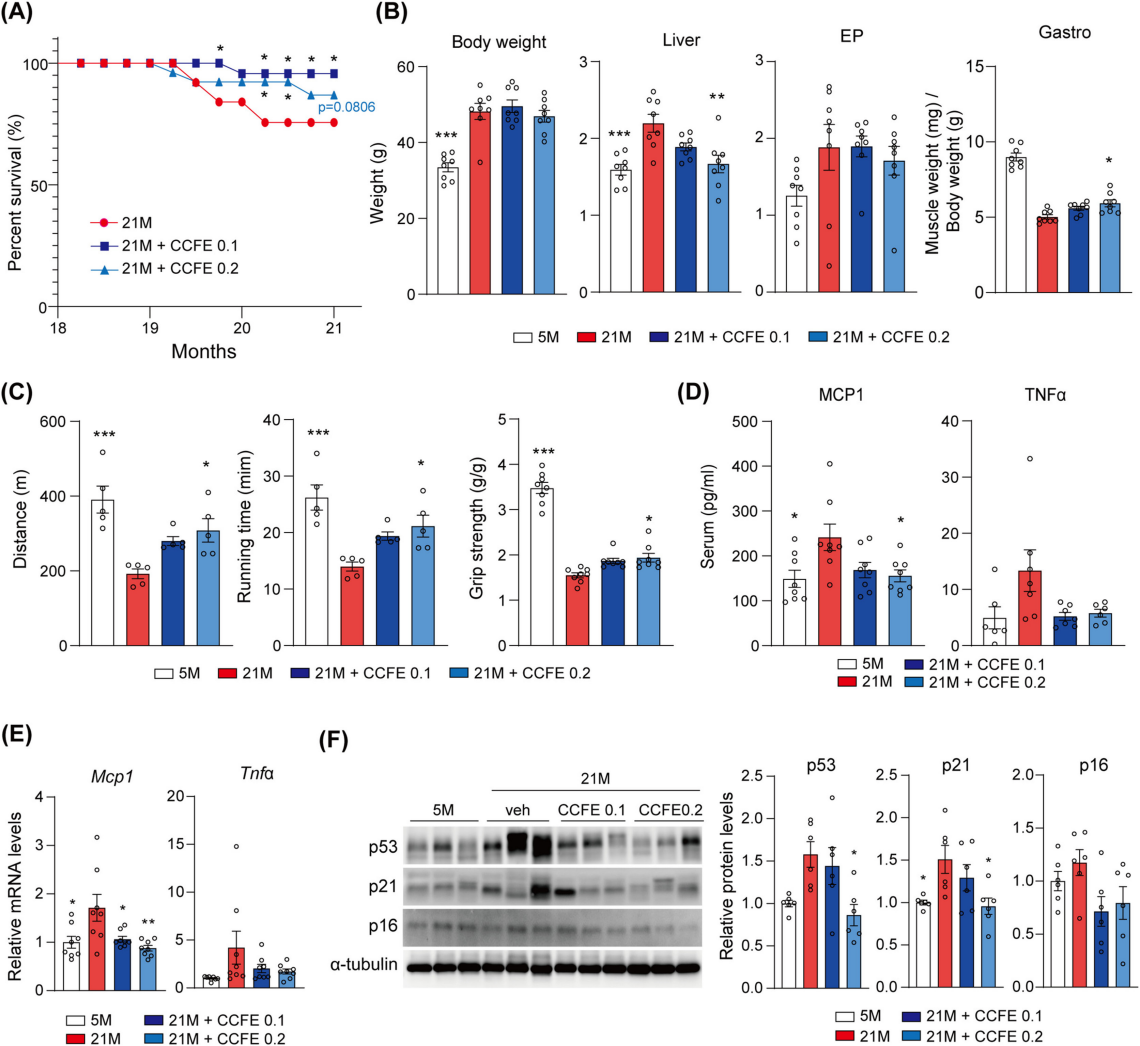
CCFE improved skeletal muscle function and suppressed cellular senescence in aged mouse skeletal muscle. (A) Comparison of survival rates among 21 months (21 M), 21 M + 0.1% CCFE (21 M + CCFE 0.1) and 21 M + 0.2% CCFE (21 M + CCFE 0.2) groups (*n* = 13). (B) Final body, liver and epididymal fat pad (EP) weight. Skeletal muscle weight (gastrocnemius) was normalized to the body weight. (C) Running distance and treadmill test time (*n* = 5 per group, left and middle panels) and grip strength normalized to body weight (*n* = 8; right panel). (D) MCP1 and TNFα levels in serum. (E) Relative mRNA expression of *Mcp1* and *Tnf*α in mouse skeletal muscle (quadriceps). (F) Immunoblotting of p53, p21 and p16 in mouse skeletal muscle (quadriceps) and quantification graph of proteins normalized to 5 M. Data are presented as mean ± SEM. *p < 0.05, **p < 0.01, ***p < 0.001 versus 21 M.

Eighteen‐month‐old mice were fed a diet containing 0.1% or 0.2% CCFE for 3 months to investigate its effects on various ageing phenotypes and age‐related skeletal muscle loss. The survival rate was monitored throughout this period. The results indicated that the CCFE‐fed groups exhibited enhanced survival rates over the 3 months (Figure 3A). Although there were no significant changes in final body weight, reductions were observed in the weights of white adipose tissue and liver in the 0.2% CCFE diet‐fed group (Figure 3B). Notably, CCFE also increased gastrocnemius muscle mass, suggesting an improvement in body composition during ageing. Treadmill and grip strength tests were conducted to evaluate skeletal muscle mass and function. The CCFE‐fed mice exhibited enhanced running distance, time and grip strength, particularly in the 0.2% CCFE diet‐fed group (Figure 3C). The 0.2% CCFE diet also reduced the serum levels of MCP1 and TNFα in aged mice (Figure 3D). Further analysis revealed that CCFE reduced the mRNA expression of *Mcp1* and *Tnf*α in skeletal muscle in the 0.2% CCFE diet‐fed group (Figure 3E). CCFE also suppressed the expression of senescence‐related proteins such as p53, p21 and p16 (Figure 3F), indicating an improvement in skeletal muscle ageing in vivo.

### 3.4 CCFE Mitigates Skeletal Muscle Atrophy

**FIGURE 4:**
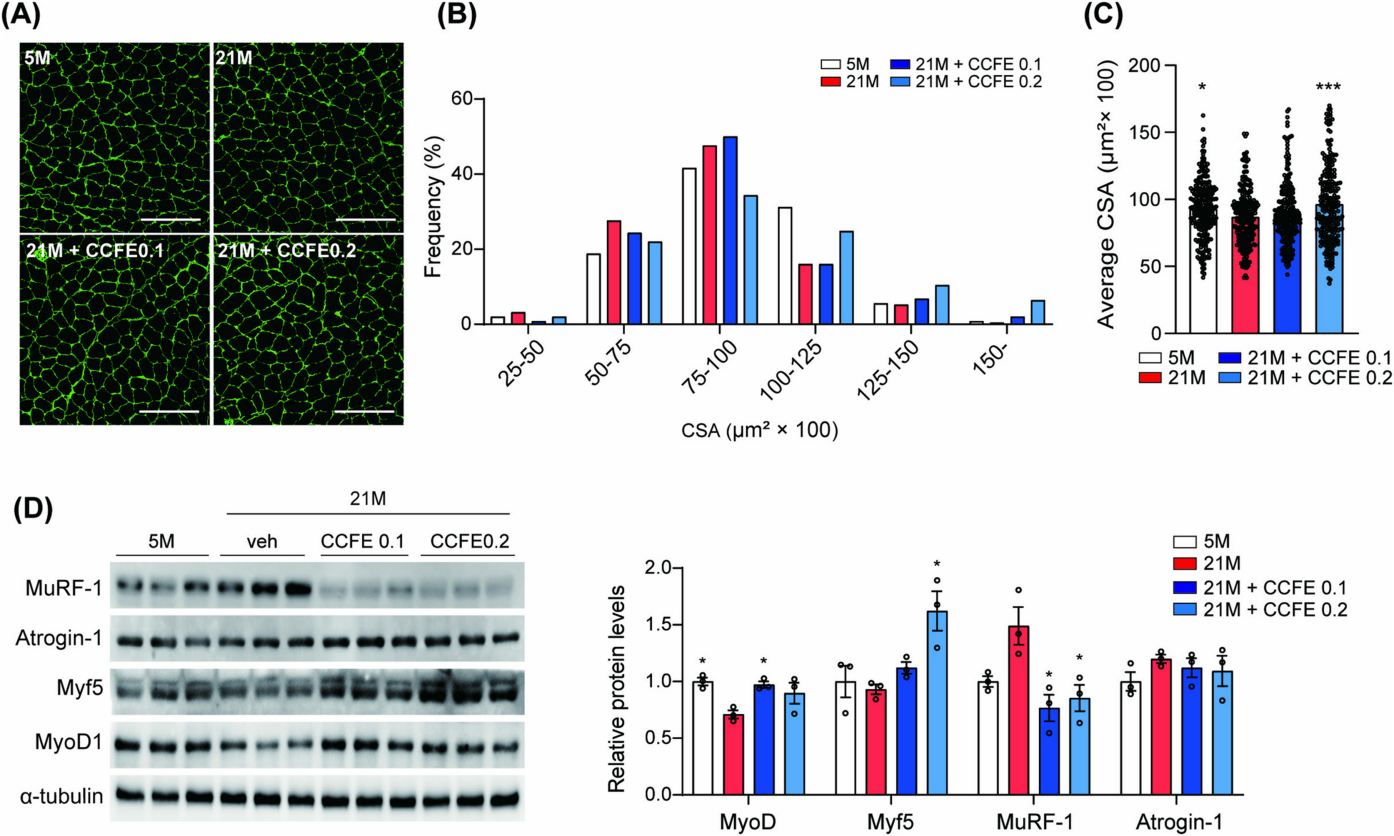
CCFE improved skeletal muscle atrophy. (A) Representative images of immunofluorescence labelling for laminin (green) in skeletal muscle (gastrocnemius) (scale bar; 200 μm). (B) Frequency of CSA and (C) average CSA of muscle fibre. (D) Protein expression level of MuRF‐2, Atrogin‐1, Myf5 and MyoD in mouse skeletal muscle and quantification graph of proteins normalized to 5 M (*n* = 3). Data are presented as mean ± SEM. *p < 0.05, ***p < 0.001 versus 21 M.

To investigate the effects of CCFE on skeletal muscle atrophy, we assessed the cross‐sectional area of the gastrocnemius muscle using immunofluorescence staining. A significant increase in cross‐sectional area was observed in the 0.2% CCFE diet‐fed group compared to the 21 M control group (Figure 4A–C). Concurrently, CCFE reduced the protein expression of the E3 ubiquitin ligases MuRf1, which regulates ubiquitin‐mediated protein degradation, and increased the protein expression of myogenesis‐related markers MyoD and Myf5 (Figure 4D). These findings suggest that CCFE inhibits excessive protein degradation by reducing E3 ubiquitin ligase activity.

### 3.5 CCFE Ameliorates Mitochondrial Dysfunction in Aged Mouse Skeletal Muscle

**FIGURE 5:**
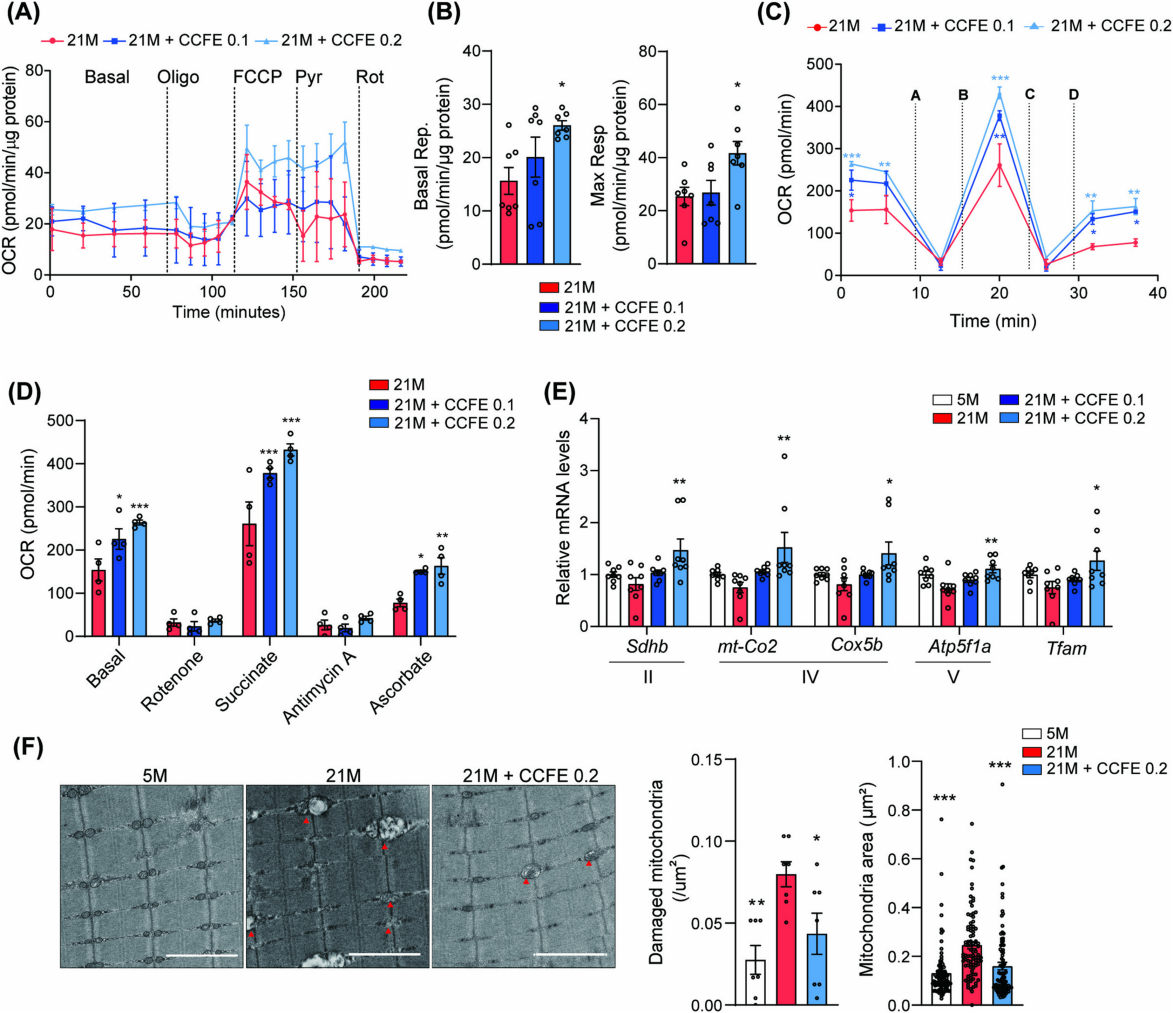
CCFE improved mitochondrial function in aged skeletal muscle. (A) OCR measurement of isolated EDL myofibres following the sequential treatment of each reagent. (B) Quantification of basal and maximal respiration of isolated EDL myofibers. (C) OCR measured using an electron flow assay of mitochondria isolated from gastrocnemius muscle (A: 2‐μM rotenone, B: 10‐mM succinate, C: 4‐μM antimycin A, D: 100‐mM ascorbate + 0.2‐mM TMPD). (D) Quantitative graph of OCR measurement of isolated mitochondria. (E) Relative mRNA levels of *Sdnb*, *mt‐Co2*, *Cox5b*, *Atp5f1a* and *Tfam* in mouse skeletal muscle. (F) Representative TEM images of longitudinal sections of mouse skeletal muscle (gastrocnemius) (scale bar: 2 μm) and quantified mitochondria area and damaged mitochondria. Data are presented as mean ± SEM. *p < 0.05, **p < 0.01 versus 21 M.

Mitochondrial dysfunction, a hallmark of ageing, contributes to increased oxidative stress and reduced exercise capacity owing to decreased ATP production [20, 21]. Therefore, the OCR in the isolated EDL muscle fibres was measured to investigate the preventive effect of CCFE on mitochondrial function decline. Both basal and maximum respiratory capacities were significantly increased in the aged 0.2% CCFE diet‐fed mice (Figure 5A,B). Additionally, an electron flow assay was conducted on mitochondria isolated from the gastrocnemius muscle to elucidate the effect of CCFE on mitochondrial function. Mitochondria were sequentially exposed to rotenone, succinate, antimycin A and ascorbate (with TMPD), along with pyruvate, malate and FCCP, and OCR was measured. CCFE increased basal OCR and OCR in the presence of succinate and ascorbate, indicating enhanced complex II and IV respiration, respectively (Figure 5C,D). Moreover, mRNA levels of mitochondrial complex genes were increased in 0.2% CCFE diet‐fed mice (Figure 5E). TEM analysis of aged skeletal muscle sections showed the presence of abnormal mitochondria. However, CCFE supplementation improved mitochondrial abnormalities (Figure 5F). These findings indicate that CCFE enhances skeletal muscle function by improving mitochondrial respiration and reducing the accumulation of damaged mitochondria.

### 3.6 CCFE Restored Autophagic Flux in Aged Mouse Skeletal Muscle

**FIGURE 6:**
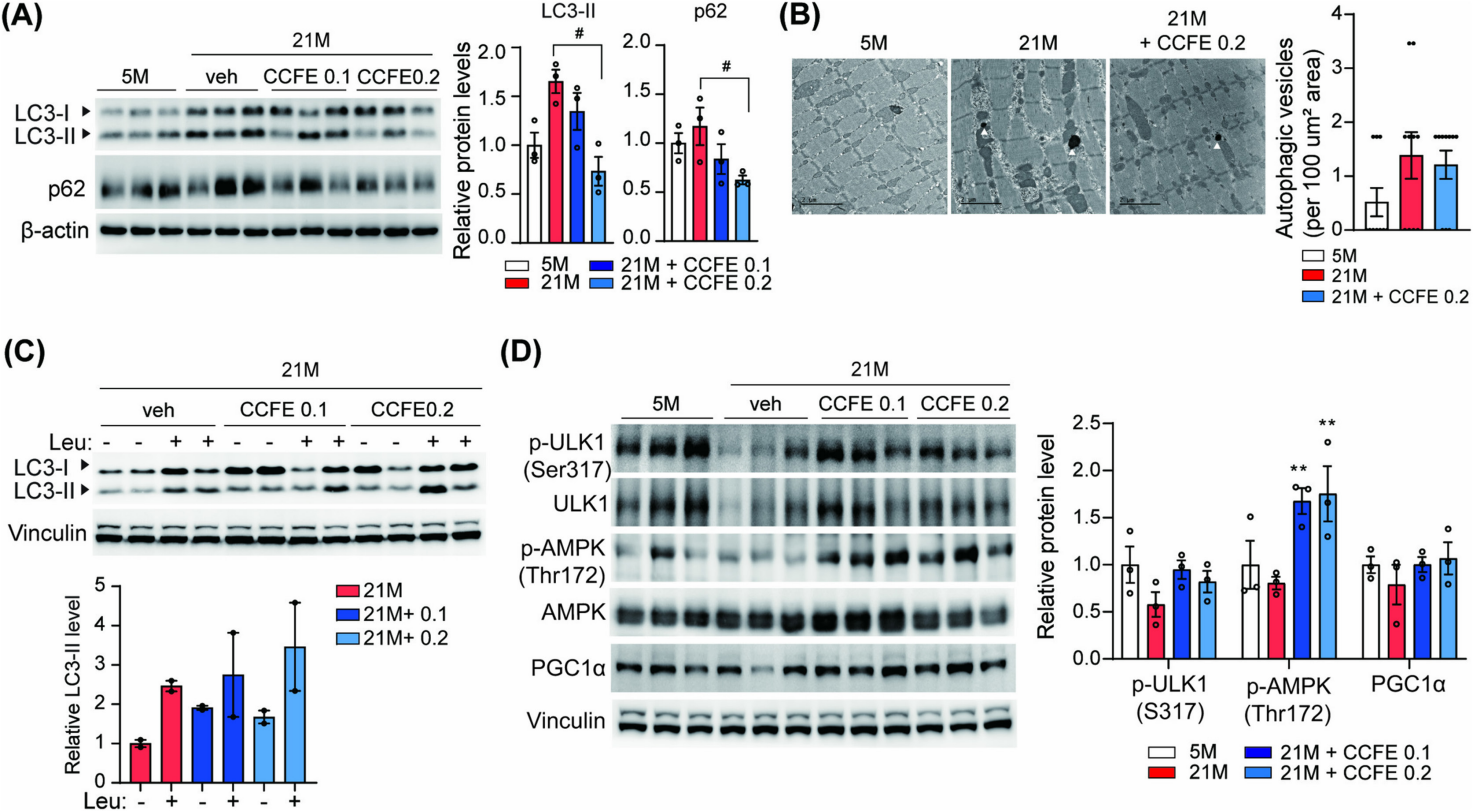
CCFE restored autophagic flux in aged skeletal muscle. (A) LC3 and p62 protein levels in gastrocnemius muscle and relative protein levels of LC3‐II and p62 normalized to 5 M. (B) Representative TEM images of the mouse gastrocnemius muscle (arrow, autophagic vesicle; scale bar: 2 μm). (C) Protein expression of LC3 in gastrocnemius muscle (17 h starvation) in the presence or absence of leupeptin (30 mg/kg, 4 h) and the graph of relative protein level. (D) Protein levels of p‐ULK1 (S317), p‐AMPK (T172), ULK1, AMPK and PGC1α in gastrocnemius muscle (17 h starvation) and the graph of phosphorylated protein levels of ULK1 and AMPK in the western blot were normalized by each total protein signal, and the relative protein levels of PGC1α were normalized by Vinculin. Data are presented as mean ± SEM. *p < 0.05, **p < 0.01, ***p < 0.001 versus 21 M.

To explore the major pathways affected by CCFE in aged skeletal muscle, we performed mRNA‐seq analysis on the tibialis anterior (TA) muscles from the 5 M, 21 M control group, and 21 M + 0.2% CCFE group. We identified 232 differentially expressed genes (DEGs) between the 21‐month‐old control group and the group treated with 0.2% CCFE (|fold change| > 1.2, normalized data (log2) > 1, *p* < 0.05) (Figure S5A,B). Gene ontology analysis revealed that the three most enriched biological processes related to autophagy—protein refolding, autophagosome assembly and autophagy—involved a total of 11 autophagy‐related genes significantly altered by CCFE treatment (Figure S5C,D). These results suggest that CCFE significantly influences autophagy activity in muscle tissue. To verify if CCFE regulates autophagy flux in aged muscle, we assessed the protein expression of LC3 and p62. Increased levels of LC3 and p62 proteins were observed in aged skeletal muscles, whereas young and CCFE‐fed groups exhibited relatively lower protein levels than those exhibited by the aged control group (Figure 6A). Additionally, TEM images revealed a greater abundance of autophagosomes in aged mice than that in the young and 0.2% CCFE diet‐fed groups (Figure 6B). Because the accumulation of these proteins often indicates blocked autophagic flux, we examined whether the increased protein levels were due to autophagy activation or autophagic flux inhibition. Autophagic flux was measured using leupeptin, an inhibitor of autophagosome‐lysosome fusion, in both young and aged mice. A decrease in autophagic flux was observed in aged skeletal muscle compared to that in young mouse skeletal muscle (Figure S6). To determine the restorative effect of CCFE on autophagic flux in aged skeletal muscles, autophagic flux in the CCFE‐fed group was examined by comparing LC3‐II levels in the absence and presence of leupeptin. CCFE significantly increased LC3‐II levels upon treatment with leupeptin, suggesting that CCFE restored autophagic flux (Figure 6C). We also examined the AMPK activity, and results indicated that CCFE significantly enhanced the phosphorylation of AMPK and ULK1 (Ser317) in aged skeletal muscles, consistent with in vitro results (Figure 6D).

### 3.7 CCFE Restores Autophagic Flux by Inhibiting EP300 Acetyltransferase Activity

**FIGURE 7:**
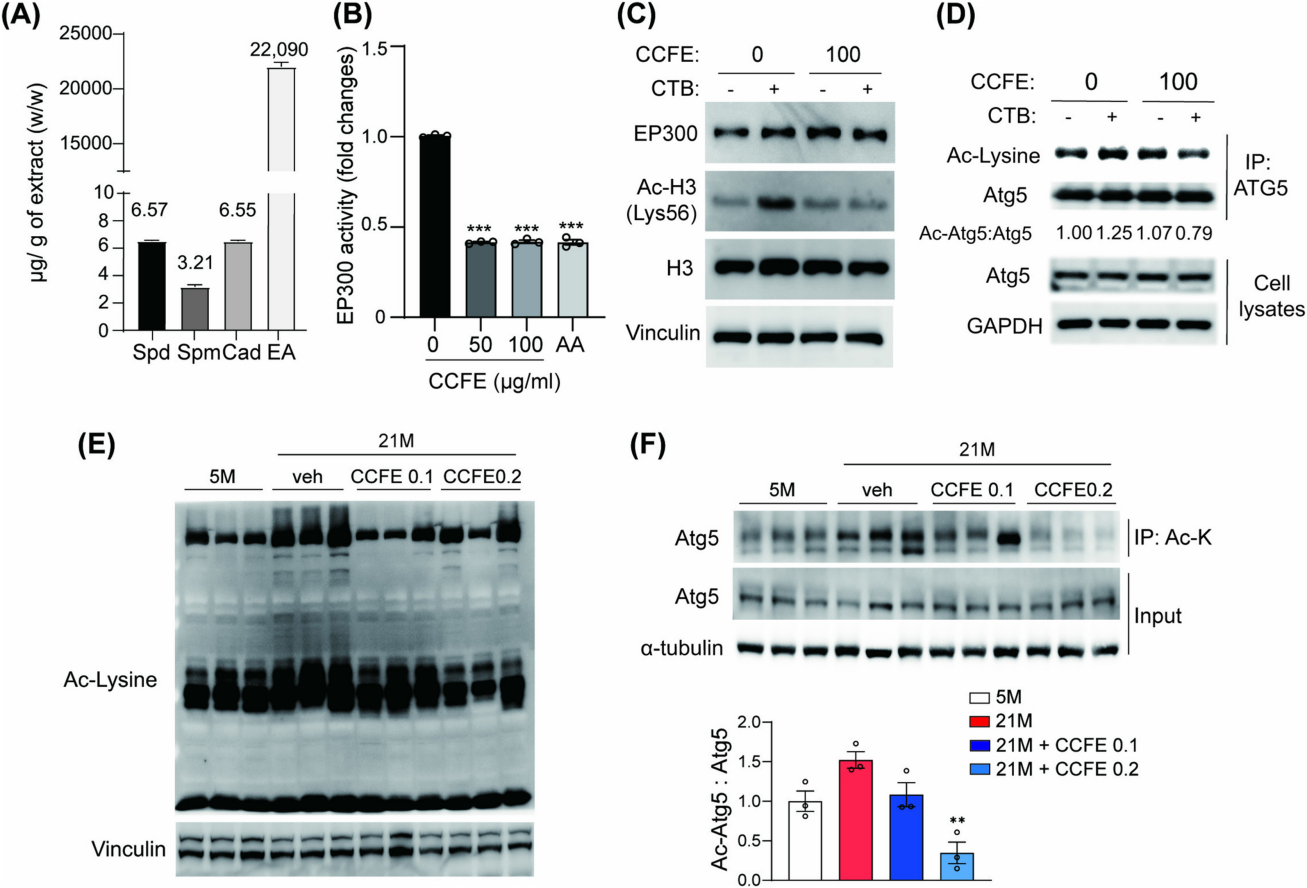
CCFE restored autophagy activity by suppressing EP300 activity. (A) The concentration of spermidine, spermine, cadaverine and ellagic acid in CCFE measured using LC‐MS/MS. (B) EP300 acetyltransferase activity of CCFE (50–100 μg/mL) and anacardic acid (AA; negative control, 100 μM). (C) Immunoblot analysis of EP300, Ac‐H3 (K56), and H3 in 293T cells treated with CCFE (100 μg/mL, 24 h) in the presence or absence of CTB (EP300 activator, 50 μM, 6 h). (D) Acetylated Atg5 level in 293T cells treated with CCFE (100 μg/mL, 24 h) or CTB (50 μM, 6 h) determined using immunoprecipitation with an acetyl‐lysine antibody. (E) Immunoblot image of total acetylated proteins in skeletal muscle. (F) Acetylated Atg5 level in skeletal muscle of 5 M, 21 M, 21 M + 0.1% CCFE and 21 M + 0.2% CCFE determined using immunoprecipitation with Atg5 antibody and the quantified graph normalized to 5 M. Data are presented as mean ± SEM. *p < 0.05, **p < 0.01, ***p < 0.001 versus 21 M.

*C. crenata* flowers have a unique fragrance, which is hypothesized to be caused by the presence of polyamines. We examined the content of these compounds in CCFE along with ellagic acid, which has been previously identified using UPLC‐MS/MS [22]. The result showed that CCFE contains substantial amounts of ellagic acid and polyamines, such as spermidine, spermine and cadaverine (Figure S7, Table S3, Figure 7A). A previous study has shown that spermidine can activate autophagy by suppressing EP300 acetyltransferase activity [23]. Therefore, we investigated whether CCFE enhances autophagy by inhibiting EP300 activity. CCFE significantly reduced EP300 activity, similar to the EP300 inhibitor, anacardic acid (Figure 7B). CCFE also inhibited the acetylation of H3 at Lys 56, which was induced by the EP300 activator, CTB (Figure 7C). EP300 suppresses autophagy by directly acetylating autophagy‐related genes [24]. In line with this, the inhibitory effect of CCFE on the acetylation of autophagy‐related genes was investigated using immunoprecipitation. CTB treatment increased the acetylation of the autophagy‐related protein Atg5, whereas CCFE restored the level of acetylated Atg5, suggesting that CCFE suppressed EP300‐induced acetylation of Atg5 (Figure 7D).

During ageing, protein acetylation in skeletal muscle is known to increase [25]. We hypothesized that CCFE restores autophagy by inhibiting the acetylation of autophagy‐related proteins. Using an acetyl‐lysine antibody, we confirmed the presence of acetylated proteins, noting that protein acetylation, which typically escalates with age, was significantly reduced in CCFE‐fed mice (Figure 7E). Furthermore, the acetylation level of Atg5, which was elevated in aged skeletal muscles, was notably decreased by CCFE (Figure 7F). These results suggest that CCFE restored autophagy by suppressing the acetylation of autophagy‐related genes in addition to regulating AMPK activity.

### 3.8 CCFE Supplementation Enhances Polyamine Metabolism and Increases Urolithin Concentrations In Vivo

**FIGURE 8:**
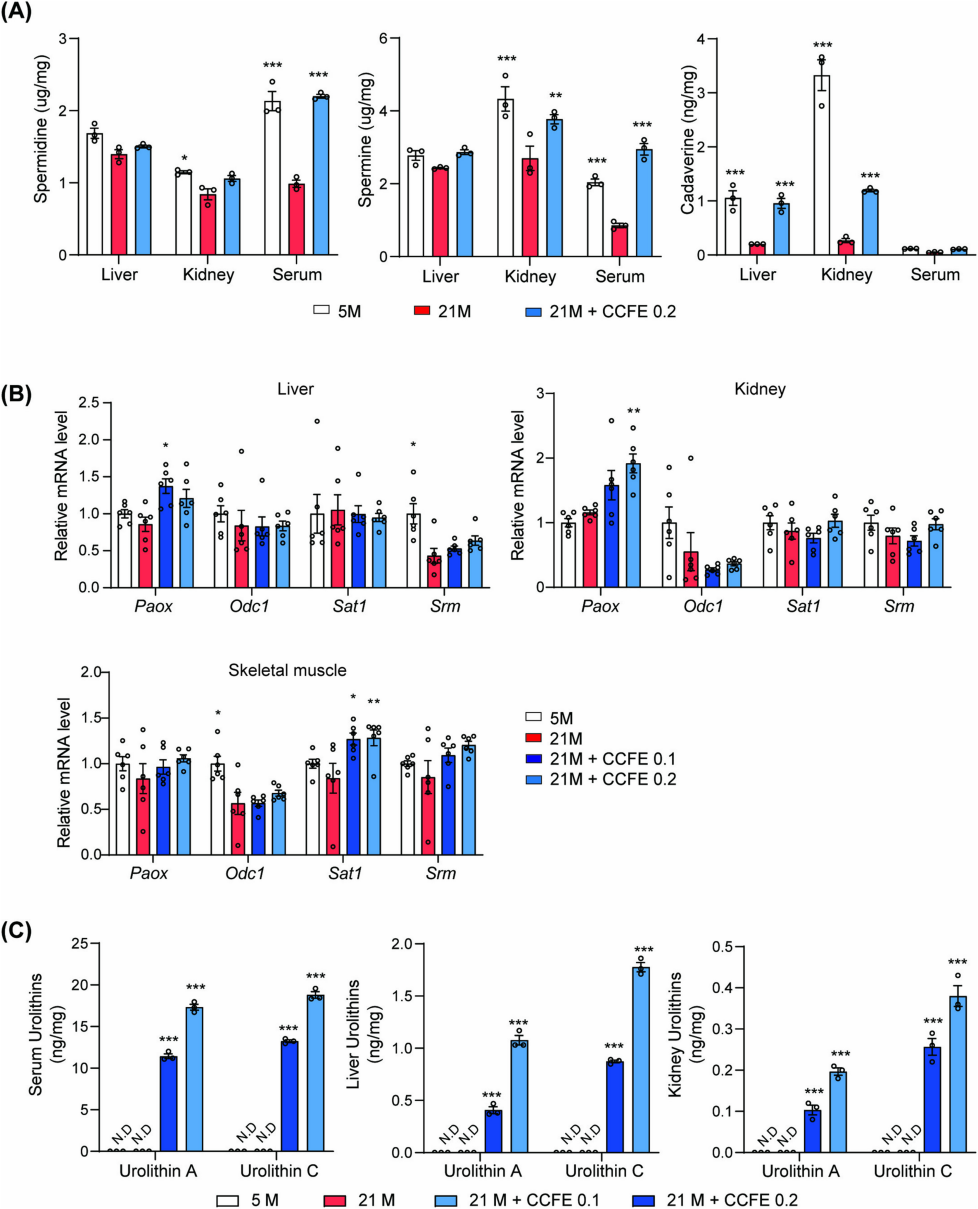
CCFE supplementation improved polyamine metabolism and increased urolithins, the metabolites of ellagic acid. (A) The levels of spermidine, spermine and cadaverine in the liver, kidney and serum of the 5 M, 21 M, CCFE and 21 M + 0.2% CCFE groups. (B) Relative mRNA level of *Paox* (polyamine oxidase), *Odc1* (ornithine decarboxylase, structural 1), *Sat1* (spermidine/spermine‐N1‐acetyltransferase) and *Srm* (spermidine synthase). (C) The amount of urolithin A and C in the liver, serum and kidney of the 5 M, 21 M, 21 M + 0.1% CCFE and 21 M + 0.2% CCFE groups. Data are presented as mean ± SEM. *p < 0.05, **p < 0.01, ***p < 0.001 versus 21 M.

Polyamines, particularly spermidine, gradually decrease during ageing, and the mRNA expression of enzymes involved in polyamine metabolism is altered in aged skeletal muscle [26]. Thus, the concentration of polyamines and the expression levels of related enzymes were measured. The UPLC‐MS/MS results revealed that CCFE supplementation elevated polyamine concentrations in the liver, kidney and serum (Figure 8A). Moreover, CCFE enhanced the mRNA expression of *Srm* and *Sat1*, which regulate polyamine metabolism (Figure 8B). These findings indicate that CCFE increased spermidine and spermine levels, thereby improving polyamine metabolism.

Ellagic acid is transformed into urolithins by the gut microbiota, and these metabolites exhibit various bioactivities [27]. The concentrations of urolithin A, B and C were measured using UPLC‐MS/MS to confirm the increase in urolithin levels following CCFE supplementation (Figure S8). In the CCFE‐fed group, urolithin A and C levels were significantly enhanced in the serum, liver and kidneys (urolithin B was not detected) (Figure 8C). These findings suggest that CCFE improves polyamine metabolism, and ellagic acid and its metabolites could contribute to the healthspan‐extending effects of CCFE.

## 4 Discussion

In this study, we identified CCFE as a novel autophagy activator, demonstrating its efficacy through various assays. Subsequently, we explored whether CCFE could contribute to healthy ageing by investigating its anti‐ageing effect on senescent cells. Cellular senescence, a hallmark of ageing, is characterized by various phenotypes, including increased SA‐β‐gal activity, damaged mitochondria and the presence of SASP [20]. It is well documented that inhibiting cellular senescence or selectively removing senescent cells suppresses ageing and extends lifespan [S10]. Previous studies have demonstrated that autophagy activation suppresses cellular senescence, while the inhibition of autophagy‐related gene expression using siRNA exacerbates cellular senescence [28, 29]. Our findings indicate that CCFE effectively inhibits cellular senescence and maintains autophagic flux; these effects were diminished in siAtg5 cells, where autophagy was compromised. These findings suggest that the enhanced autophagic flux induced by CCFE can delay cellular senescence.

We next investigated whether CCFE could extend the healthspan and delay muscle ageing in aged mice. Our findings indicate that CCFE significantly increased the survival rate of aged mice up to 21 months and enhanced their running distance, time and grip strength. Additionally, CCFE increased muscle mass compared to controls. These benefits were accompanied by reductions in ageing hallmarks, especially CCFE‐suppressed cellular senescence markers and inflammation in aged skeletal muscle. Furthermore, CCFE significantly enhanced mitochondrial function, as evidenced by increased OCR and improved mitochondrial quality. We investigated whether CCFE activates autophagy in the muscle tissue of aged mice injected with leupeptin [30]. The results indicated a significant increase in autophagic flux in aged muscle tissue. Although our in vivo results do not directly link muscle ageing with autophagic activity, prior research has highlighted the interplay between autophagy and several ageing hallmarks, along with the dependency of several anti‐ageing interventions on autophagy [S11]. Our findings regarding CCFE's impact on autophagic flux in aged skeletal muscles provide valuable insights into how enhancing autophagy could potentially mitigate skeletal muscle ageing.

We sought to determine the mechanism of autophagy activation of CCFE. LC‐MS analysis revealed that CCFE is rich in polyamines such as spermidine and spermine, which are known to activate autophagy by inhibiting EP300 acetyltransferase, a negative regulator of autophagy that directly acetylates ATG5, ATG7 and ATG12 [24, 31]. CCFE significantly inhibited EP300 acetyltransferase activity in muscle tissue. These results suggest that increased protein acetylation in aged skeletal muscle can negatively affect autophagy and indicate the possibility that EP300 inhibition can restore autophagic flux in aged skeletal muscle. Additionally, the levels of polyamines are reduced, and the expression of genes involved in polyamine metabolism is altered in aged muscle. [26, 32]. CCFE supplementation not only increased the levels of spermidine, spermine and cadaverine but also altered the expression of genes encoding enzymes involved in polyamine metabolism, including *Sat1* and *Srm*. SAT1 catalyses the transfer of an acetyl group from acetyl‐CoA to spermidine and spermine [33]. SAT1 regulates polyamine metabolic flux by increasing the activity of key biosynthetic enzymes [34]. Additionally, SRM restoration may contribute to increased spermidine levels. Overall, these findings suggest that CCEE provides exogenous polyamines and enhances polyamine metabolic flux by upregulating the expression of related genes.

Ellagic acid, the most abundant component of CCFE, activates autophagy by enhancing AMPK activity and suppressing the mTOR pathway [35]. Ellagic acid prolonged the lifespan of *D. melanogaster* and suppressed cellular senescence, indicating its contribution to the autophagy‐activating and anti‐ageing effects of CCFE [36, 37]. Urolithins are natural food metabolites produced by the gut microbiome from ellagic acid [27]. CCFE supplementation increased urolithin A and C levels in aged mice. Urolithin A has been reported to extend the lifespan of *C. elegans* and enhance muscle function by activating mitophagy [38]. Studies on the biological activity of urolithin C have not been fully conducted; however, a previous study suggested that urolithin C increases MAPK1/ERK activity, indicating its potential involvement in various biological processes [39].

In conclusion, CCFE suppresses skeletal muscle ageing by preventing mitochondrial dysfunction and restoring autophagic flux in aged mice. CCFE exerted this effect by modulating AMPK and EP300 acetyltransferase activity, which enhanced autophagy. These findings suggest the potential of CCFE as a therapeutic agent for extending healthspan and mitigating sarcopenia, providing a basis for future clinical trials.

## Ethics Statement

All animal experiments were approved by the Animal Care and Use Committee of the Korea Food Research Institute (KFRI‐M‐19030).

## Conflicts of Interest

The authors declare no conflicts of interest.

## Supporting information


**Table S2.** Supporting Information.

**Figure S1.** Autophagy activator screening of 493 natural products using Cyto‐ID. (A) Heatmap showing autophagy activity of 493 natural products normalized to the CQ‐treated cells (10 nM). (B) Heatmap showing autophagy activity of 493 natural products normalized to the RM + CQ‐treated cells. Detailed information on the 493 natural products is provided in Table S2.
**Figure S2.** Autophagic flux analysis of 24 selected natural products. Immunoblot images of Huh7 cells treated with 24 natural products (100 μg/mL) or RM (100 nM) for 24 h in the presence or absence of CQ (10 μM).
**Figure S3.** Effect of CCFE on cell viability and autophagy flux. (A) Cell viability of HeLa and C2C12 cells treated with *Castanea crenata* flower extract **(**CCFE, 25–200 μg/mL). *** *p* < 0.001 vs. control. (B) The ratio of LC3II to LC3I in C2C12 cells treated with CCFE (50–100 μg/mL) or rapamycin (RM, 50 nM) for 2 h in the presence or absence of bafilomycin A1 (Baf, 25 nM, 2 h). (C) C2C12 cells transiently transfected with mCherry‐EGFP‐LC3 and treated with Baf (25 nM), CCFE (100 μg/mL), or Torin 1 (200 nM) for 2 h. After cell nucleus staining with DAPI, cells were examined for autophagic flux using confocal microscopy (scale bar: 20 μm). (D) Number of red and yellow puncta examined in the merged images (*n* = 3). * *p* < 0.005 vs. control (yellow dot); # *p* < 0.005 vs. control (red dot). Data are presented as mean ± SD.
**Figure S4.** The inhibitory effect of CCFE on cellular senescence in human skeletal muscle cells and siAtg5 C2C12 cells (A) Senescence‐associated β‐galactosidase (SA β‐gal) staining images (scale bar; 100 μm) of passage 6 (p6) and passage 12 (p12) human skeletal muscle myoblasts (HSMM) cells. p12 cells treated with CCFE (50–100 μg/mL) or RM (50 nM) for 24 h and the quantified SA β‐gal‐positive cell. (B) Protein expression of p21 and p16 in p6 and p12 HSMM cells treated with CCFE (50–100 μg/mL) or RM (50 nM) for 24 h and the quantification graph of proteins normalized to p12 control cells (*n* = 3). * *p* < 0.05, * *p* < 0.01, *** *p* < 0.001 vs. p12 HSMM control cells. (C) Protein expression of Atg5 and p16 in normal and etoposide‐induced senescent siNC or si*Atg5* C2C12 cells treated with CCFE (50–100 μg/mL) or rapamycin (RM, 50 nM) for 2 h and the quantification graph of proteins normalized to normal siNC cells (*n* = 3, * *p* < 0.05, siNC vs. si*Atg5* cells, t‐test). The data are presented as the mean ± SD of triplicate experiments.
**Figure S5.** RNA sequencing analysis of mouse skeletal muscle (n = 3) (A) Volcano plot of DEGs between 21 M and 21 M + 0.2% CCFE (21M+CCFE 0.2, |Fold change| > 1.2, *p* < 0.05) represented as log10 (p‐value) versus log2 (fold change). (B) Heatmap of DEGs between 21 M and 21 M + 0 CCFE0.2 (|Fold change| > 1.2, Normalized data (log2) > 1, *p* < 0.05). (C) The top 12 significant GO terms of biological processes (BP), cellular component (CC), and molecular function (MF) associated with the identified DEGs between 21 M and 21 M + CCFE0.2. (D) Autophagy‐related DEGs between 21 M and 21 M + CCFE0.2 in aged muscle.
**Figure S6.** Autophagic flux of skeletal muscle in young and old mice. Protein expression of LC3 in skeletal muscle of 5‐month‐old (5 M) and 21‐month‐old mice (21 M) treated with phosphate‐buffered saline or leupeptin (30 mg/kg, intraperitoneal injection, 4 h).
**Figure S7.** Identification of spermidine, spermine, cadaverine, and ellagic acid in CCFE.
**Figure S8.** UPLC‐MS/MS analysis of urolithin A, B, and C. Representative chromatograms of urolithin A (top), B (middle), and C (bottom).
**Table S1.** Primer sequences used in this study
**Table S3.** LOD and LOQ of LC–MS/MS analysis
