# Supplementary material for: A Natural Autophagy Activator Castanea crenata Flower Alleviates Skeletal Muscle Ageing
Source: J Cachexia Sarcopenia Muscle. 2025 Jan 28;16(1):e13710. doi: 10.1002/jcsm.13710 (PMC11773338; doi:10.1002/jcsm.13710)
Supplement: Supplementary file 2 — Figure S1. Autophagy activator screening of 493 natural products using Cyto‐ID. (A) Heatmap showing autophagy activity of 493 natural products normalized to the CQ‐treated cells (10 nM). (B) Heatmap showing autophagy activity of 493 natural products normalized to the RM + CQ‐treated cells. Detailed information on the 493 natural products is provided in Table S2. Figure S2. Autophagic flux analysis of 24 selected natural products. Immunoblot images of Huh7 cells treated with 24 natural products (100 μg/mL) or RM (100 nM) for 24 h in the presence or absence of CQ (10 μM). Figure S3. Effect of CCFE on cell viability and autophagy flux. (A) Cell viability of HeLa and C2C12 cells treated with Castanea crenata flower extract (CCFE, 25–200 μg/mL). *** p < 0.001 vs. control. (B) The ratio of LC3II to LC3I in C2C12 cells treated with CCFE (50–100 μg/mL) or rapamycin (RM, 50 nM) for 2 h in the presence or absence of bafilomycin A1 (Baf, 25 nM, 2 h). (C) C2C12 cells transiently transfected with mCherry‐EGFP‐LC3 and treated with Baf (25 nM), CCFE (100 μg/mL), or Torin 1 (200 nM) for 2 h. After cell nucleus staining with DAPI, cells were examined for autophagic flux using confocal microscopy (scale bar: 20 μm). (D) Number of red and yellow puncta examined in the merged images (n = 3). * p < 0.005 vs. control (yellow dot); # p < 0.005 vs. control (red dot). Data are presented as mean ± SD. Figure S4. The inhibitory effect of CCFE on cellular senescence in human skeletal muscle cells and siAtg5 C2C12 cells (A) Senescence‐associated β‐galactosidase (SA β‐gal) staining images (scale bar; 100 μm) of passage 6 (p6) and passage 12 (p12) human skeletal muscle myoblasts (HSMM) cells. p12 cells treated with CCFE (50–100 μg/mL) or RM (50 nM) for 24 h and the quantified SA β‐gal‐positive cell. (B) Protein expression of p21 and p16 in p6 and p12 HSMM cells treated with CCFE (50–100 μg/mL) or RM (50 nM) for 24 h and the quantification graph of proteins normalized to p12 control cells (n = 3). [file JCSM-16-e13710-s001.docx]

**Journal of Cachexia, Sarcopenia and Muscle**

**[Supplementary information]**

**A natural autophagy activator *Castanea crenata* flower alleviates skeletal muscle aging**

So-Hyun Park ^1,2^, Pyeong Geun Choi ^1,2^, Hee-Soo Kim^1,2^, Eunyoung Lee^1^, Da-Hye Lee^1,3^, Min Jung Kim ^4^, Daedong Kim^1,2^, Hyo-Deok Seo ^1^, Jeong-Hoon Hahm^1^, Tae-Il Jeon^5^, Yang-Hoon Huh^6^, Jiyun Ahn ^1,2^, Tae-Youl Ha^1,2^, Chang Hwa Jung ^1,2^ *

* Correspondence to: Chang Hwa Jung, Aging and Metabolism Research Group, Korea Food Research Institute, 245, Nongsaengmyeong-ro, Iseo-myeon, Wanju_Gun, Jeollabuk-do, 55365, Republic of Korea.

E-mail address: chjung@kfri.re.kr

**Contents:**

Supplementary Figures [Figure S1-8]

Supplementary tables [Table S1,3]

Supplementary Methods

Supplementary references

**Supplementary Figure**

**
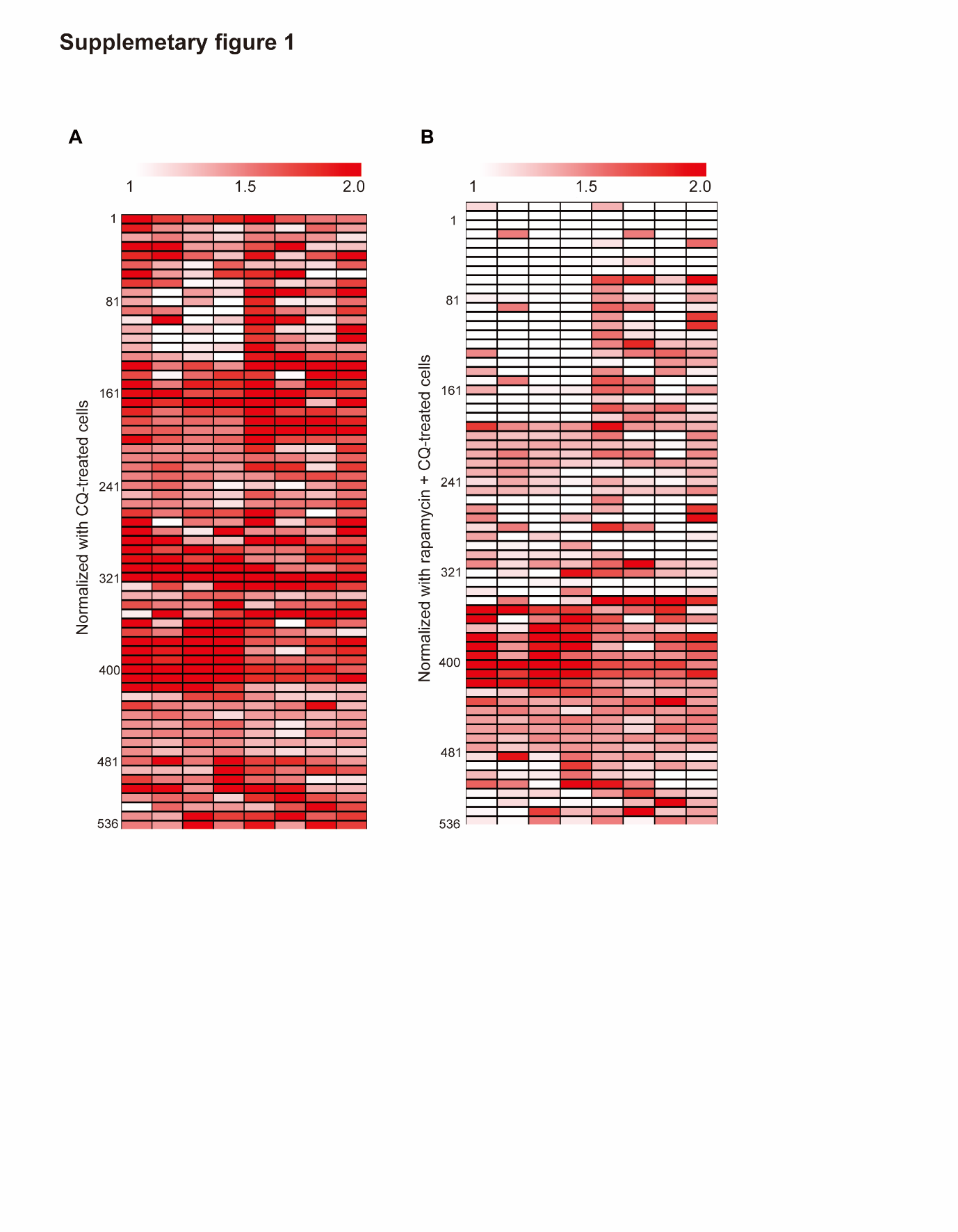
**

**Figure S1.** Autophagy activator screening of 493 natural products using Cyto-ID. (A) Heatmap showing autophagy activity of 493 natural products normalized to the CQ-treated cells (10 nM). (B) Heatmap showing autophagy activity of 493 natural products normalized to the RM + CQ-treated cells. Detailed information on the 493 natural products is provided in Table S2.


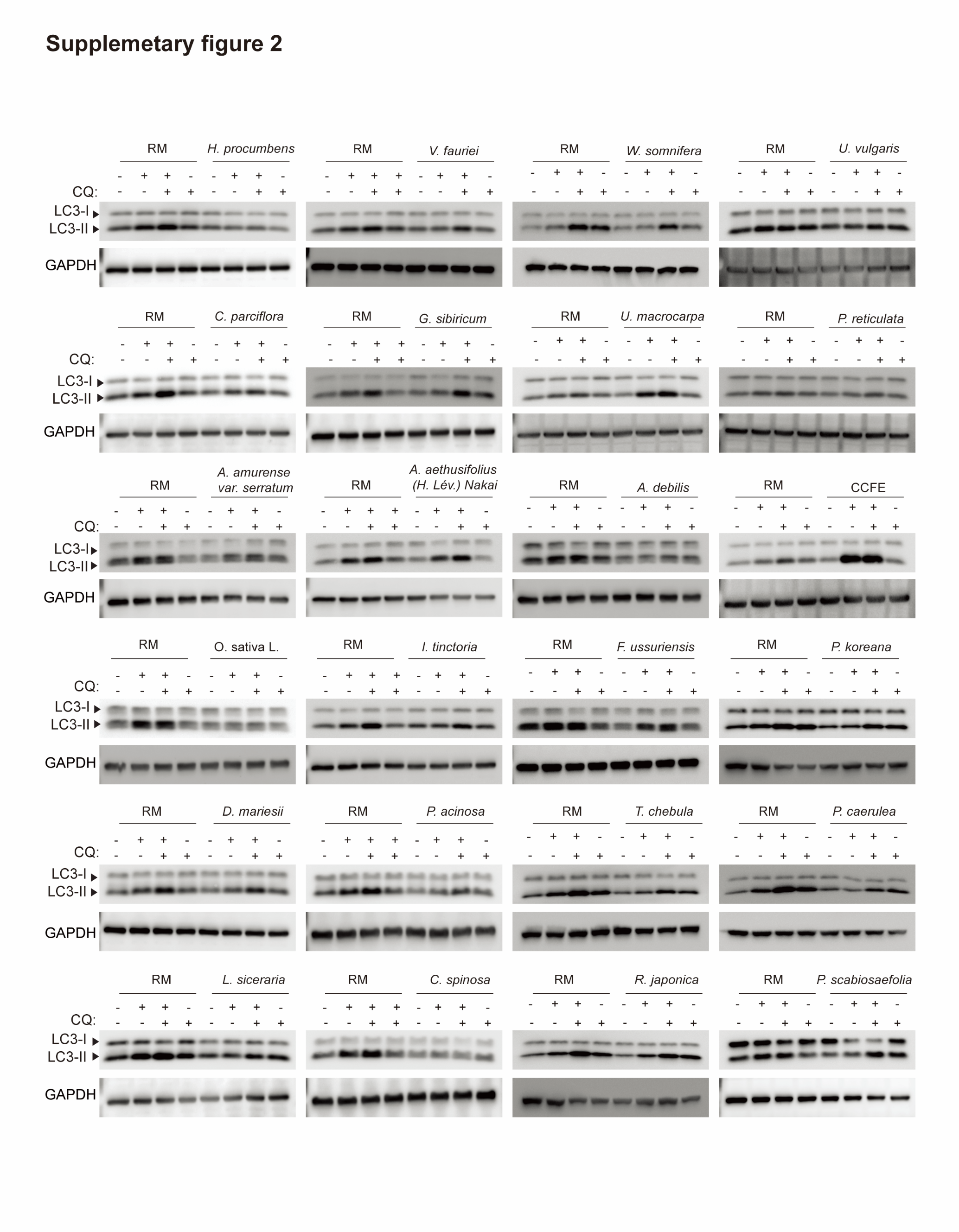


**Figure S2.** Autophagic flux analysis of 24 selected natural products**.** Immunoblot images of Huh7 cells treated with 24 natural products (100 μg/mL) or RM (100 nM) for 24 h in the presence or absence of CQ (10 μM).


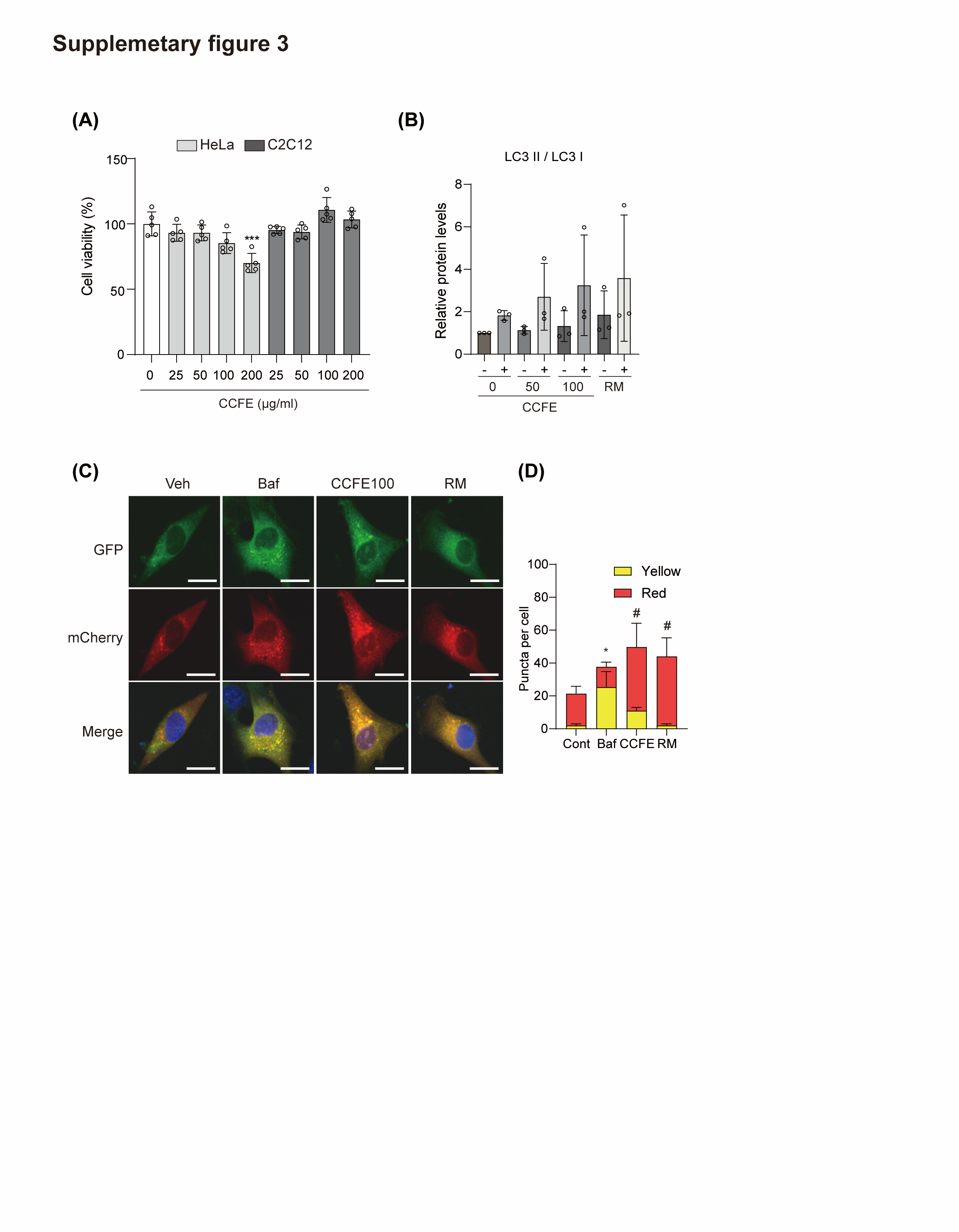


**Figure S3.** Effect of CCFE on cell viability and autophagy flux. (A) Cell viability of HeLa and C2C12 cells treated with *Castanea crenata* flower extract **(**CCFE, 25–200 μg/ml). *** *p* < 0.001 vs. control. (B) The ratio of LC3Ⅱ to LC3Ⅰ in C2C12 cells treated with CCFE (50–100 μg/ml) or rapamycin (RM, 50 nM) for 2 h in the presence or absence of bafilomycin A1 (Baf, 25 nM, 2 h). (C) C2C12 cells transiently transfected with mCherry-EGFP-LC3 and treated with Baf (25 nM), CCFE (100 μg/ml), or Torin 1 (200 nM) for 2 h. After cell nucleus staining with DAPI, cells were examined for autophagic flux using confocal microscopy (scale bar: 20 μm). (D) Number of red and yellow puncta examined in the merged images (n = 3). * *p* < 0.005 vs. control (yellow dot); # *p* < 0.005 vs. control (red dot). Data are presented as mean ± SD.

**
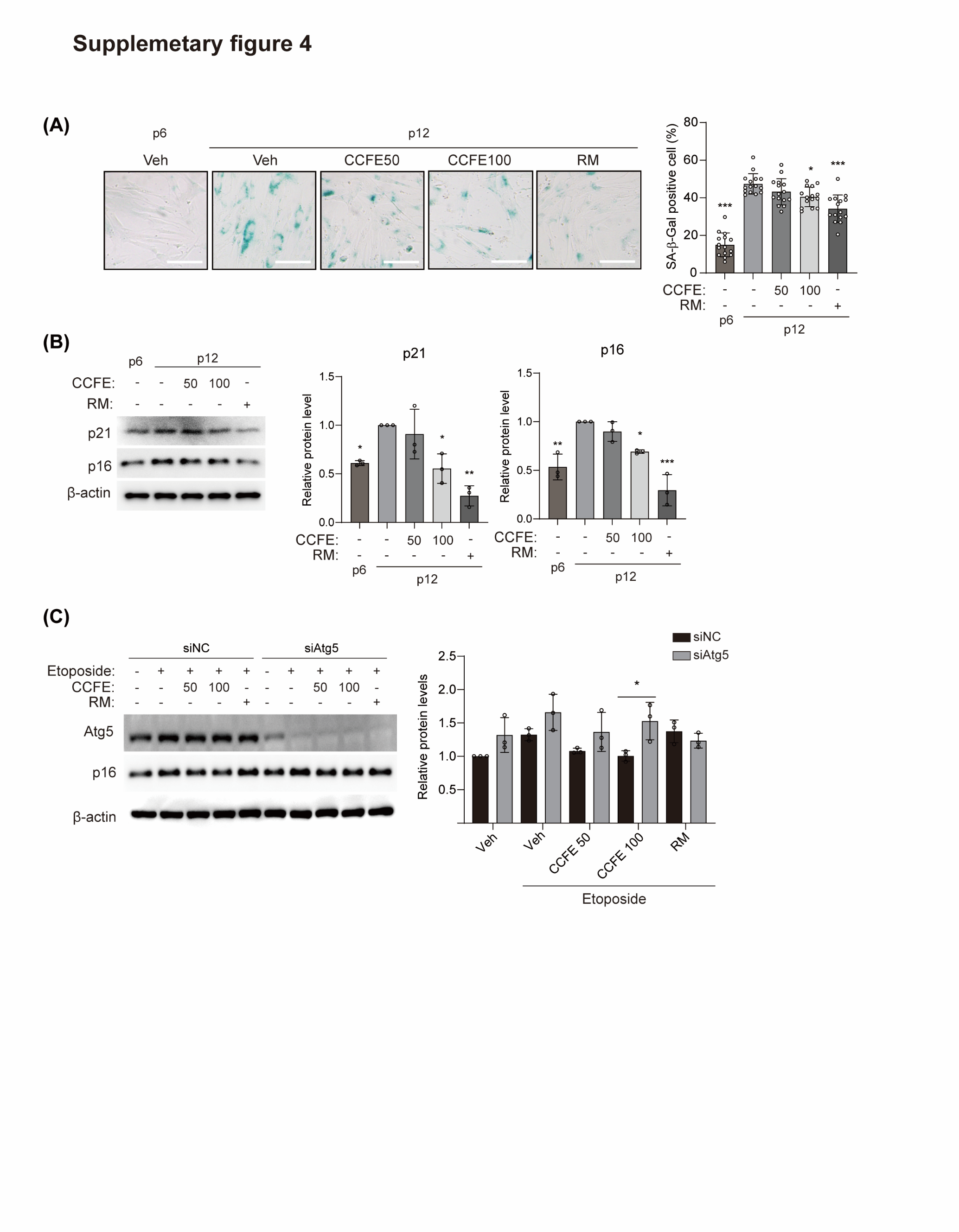
**

**Figure S4.** The inhibitory effect of CCFE on cellular senescence in human skeletal muscle cells and siAtg5 C2C12 cells (A) Senescence-associated β-galactosidase (SA β-gal) staining images (scale bar; 100 μm) of passage 6 (p6) and passage 12 (p12) human skeletal muscle myoblasts (HSMM) cells. p12 cells treated with CCFE (50–100 μg/ml) or RM (50 nM) for 24 h and the quantified SA β-gal-positive cell. (B) Protein expression of p21 and p16 in p6 and p12 HSMM cells treated with CCFE (50–100 μg/ml) or RM (50 nM) for 24 h and the quantification graph of proteins normalized to p12 control cells (n = 3). * *p* < 0.05, * *p* < 0.01, *** *p* < 0.001 vs. p12 HSMM control cells. (C) Protein expression of Atg5 and p16 in normal and etoposide-induced senescent siNC or si*Atg5* C2C12 cells treated with CCFE (50–100 μg/ml) or rapamycin (RM, 50 nM) for 2 h and the quantification graph of proteins normalized to normal siNC cells (n = 3, * *p* < 0.05, siNC vs. si*Atg5* cells, t-test). The data are presented as the mean ± SD of triplicate experiments.


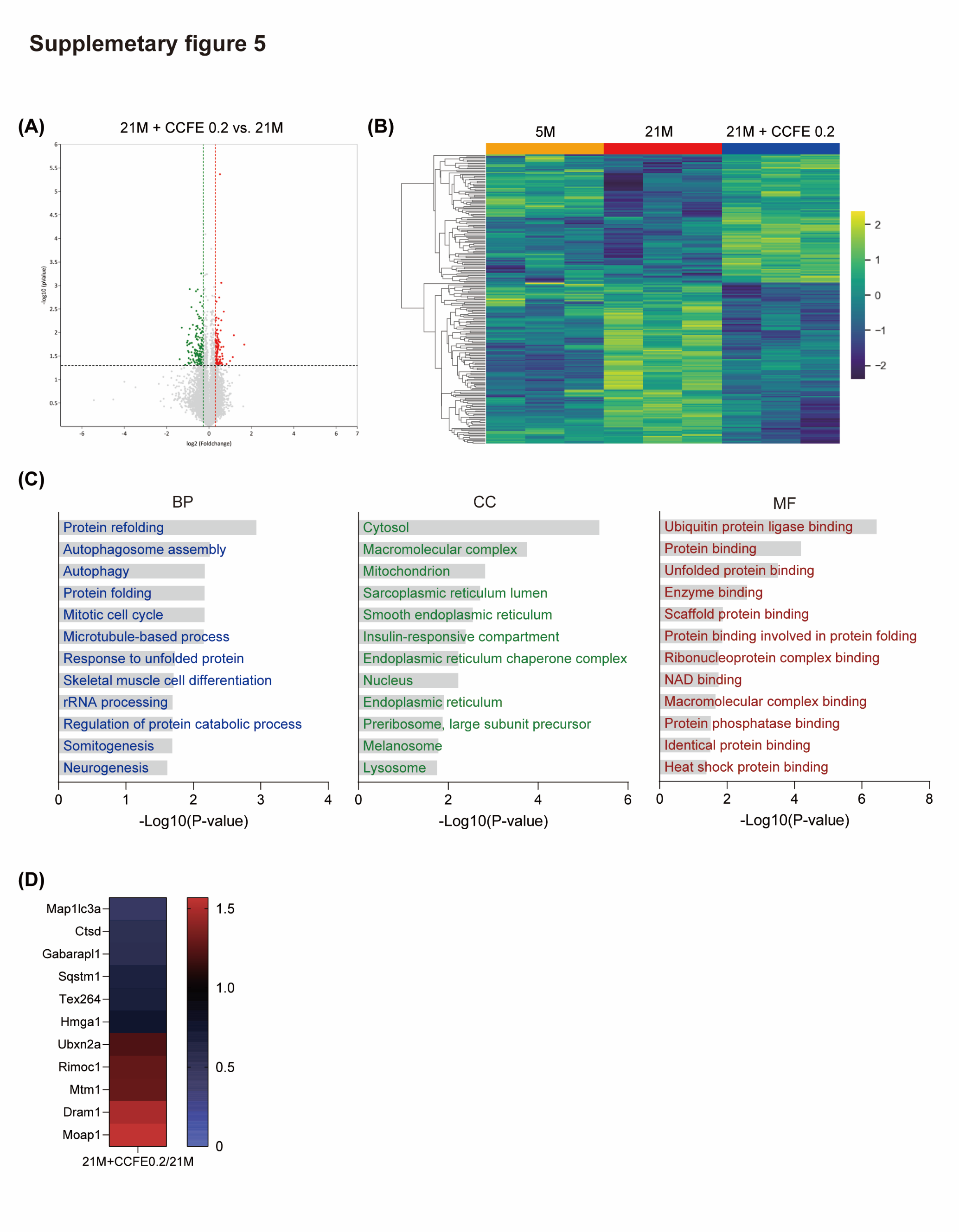


**Figure S5.** RNA sequencing analysis of mouse skeletal muscle (n = 3) (A) Volcano plot of DEGs between 21 M and 21 M + 0.2% CCFE (21M+CCFE0.2, |Fold change| >1.2, *p* < 0.05) represented as log10 (p-value) versus log2 (fold change). (B) Heatmap of DEGs between 21 M and 21 M+ CCFE0.2 (|Fold change| >1.2, Normalized data (log2)>1, *p* < 0.05). (C) The top 12 significant GO terms of biological processes (BP), cellular component (CC), and molecular function (MF) associated with the identified DEGs between 21 M and 21 M + CCFE0.2. (D) Autophagy-related DEGs between 21 M and 21 M + CCFE 0.2 in aged muscle.

**
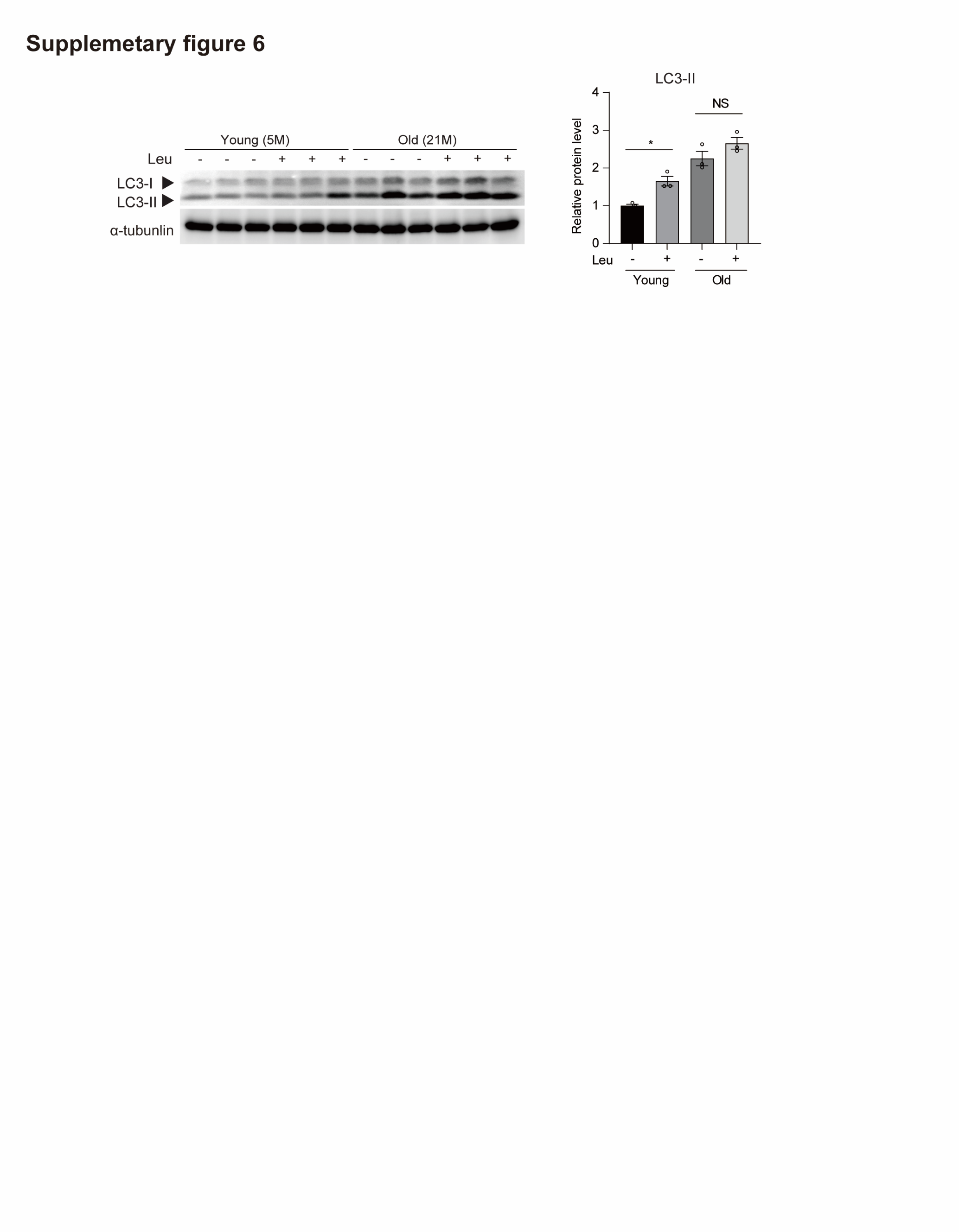
****Figure S6.** Autophagic flux of skeletal muscle in young and old mice. Protein expression of LC3 in skeletal muscle of 5-month-old (5 M) and 21-month-old mice (21 M) treated with phosphate-buffered saline or leupeptin (30 mg/kg, intraperitoneal injection, 4 h).


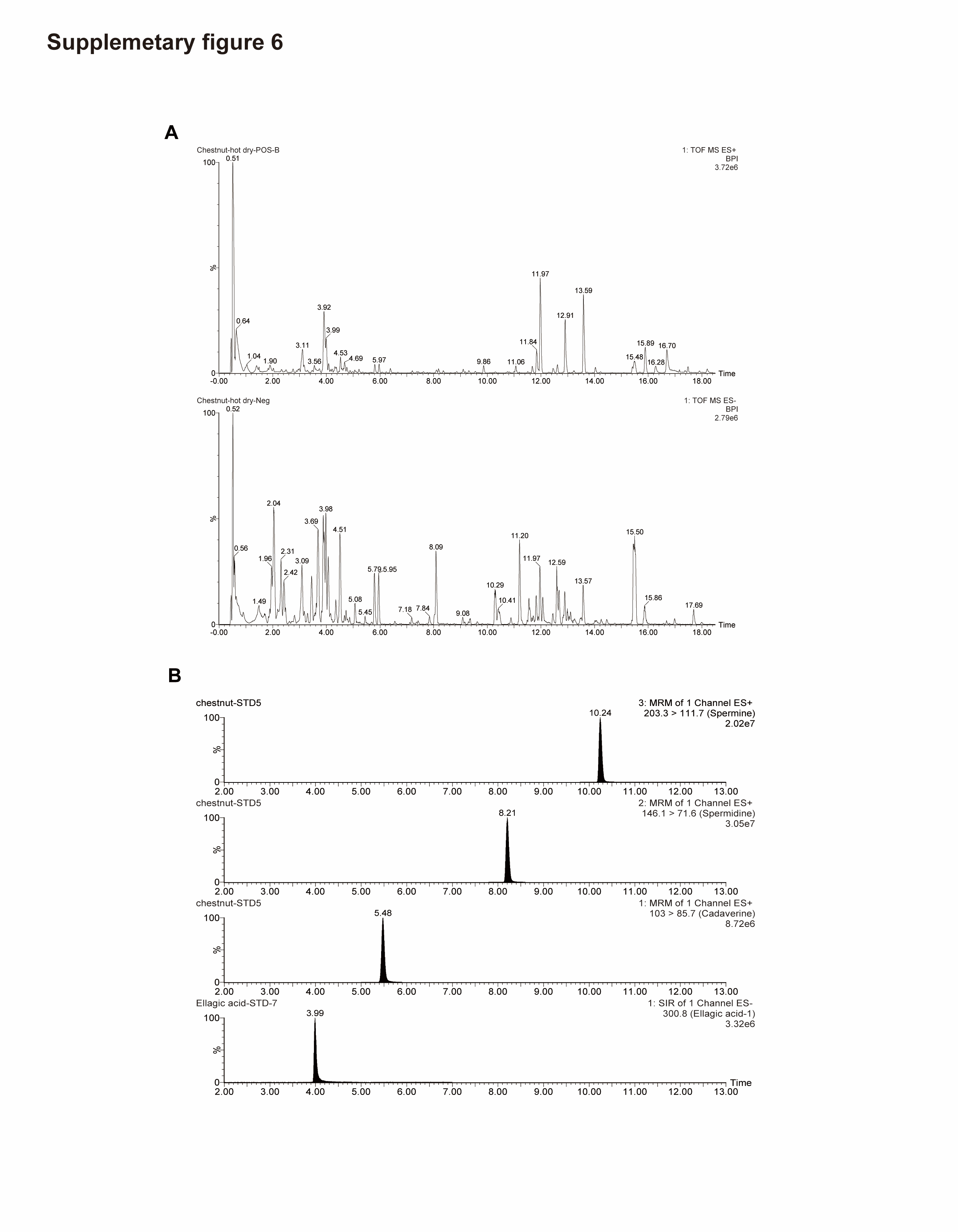


**Figure S7.** Identification of spermidine, spermine, cadaverine, and ellagic acid in CCFE.

**
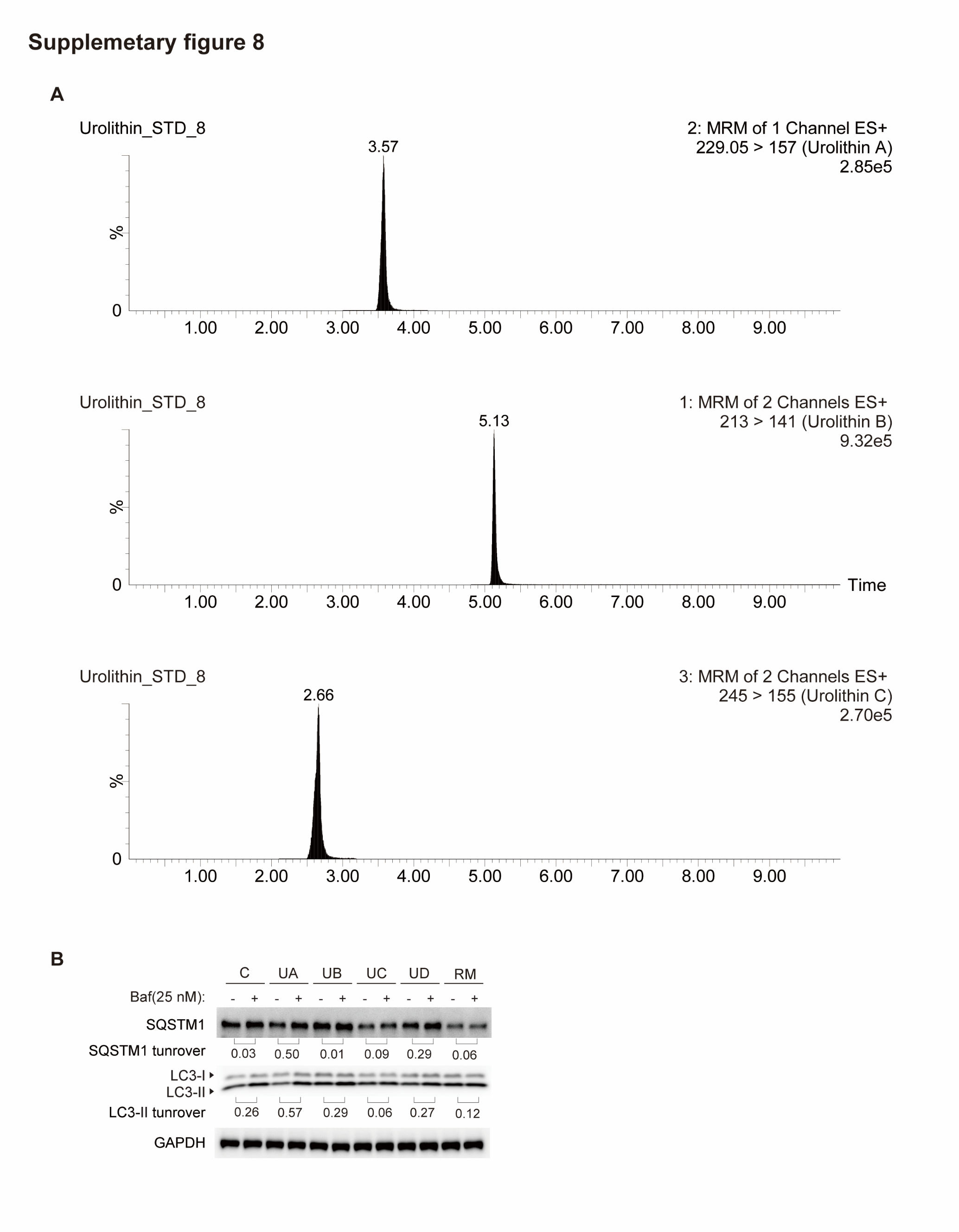
****Figure S8**. UPLC-MS/MS analysis of urolithin A, B, and C. Representative chromatograms of urolithin A (top), B (middle), and C (bottom).

| Supplementary table  Table S1 Primer sequences used in this study | | | |
| --- | --- | --- | --- |
| Genes |  | Sequences | |
| *P62* | Forward | | ATGTGGAACATGGAGGGAAGA |
|  | Reverse | | GGAGTTCACCTGTAGATGGGT |
| *MCP1* | Forward | | CCACTCACCTGCTGCTACTCA |
|  | Reverse | | TGGTGATCCTCTTGTAGCTCTCC |
| *Il1a* | Forward | | CGAAGACTACAGTTCTGCCATT |
|  | Reverse | | GACGTTTCAGAGGTTCTCAGAG |
| *Il6* | Forward | | TAGTCCTTCCTACCCCAATTTCC |
|  | Reverse | | TTGGTCCTTAGCCACTCCTTC |
| *Tnf* | Forward | | GCCACCACGCTCTTCTGCCT |
|  | Reverse | | GGCTGATGGTGTGGGTGAGG |
| *Sdhb* | Forward | | AATTTGCCATTTACCGATGGGA |
|  | Reverse | | AGCATCCAACACCATAGGTCC |
| *mt-Co2* | Forward | | ATAACCGAGTCGTTCTGCCA |
|  | Reverse | | GCTTGATTTAGTCGGCCTGG |
| *Cox5b* | Forward | | TTCAAGGTTACTTCGCGGAGT |
|  | Reverse | | CGGGACTAGATTAGGGTCTTCC |
| *Atp5f1a* | Forward | | TTTGCCCAGTTTGGTTCTGAT |
|  | Reverse | | CCCGTACACCCGCATAGATAA |
| *Tfam* | Forward | | GTCGCATCCCCTCGTCTATC |
|  | Reverse | | GCTGGAAAAACACTTCGGAATAC |
| *Paox* | Forward | | CTTCGGTGGTGTAGTGGAGC |
|  | Reverse | | TCCGATAATTCTTTCTCCCCCAG |
| *Odc1* | Forward | | GACGAGTTTGACTGCCACATC |
|  | Reverse | | CGCAACATAGAACGCATCCTT |
| *Sat1* | Forward | | GAGAACACCCCTTCTACCACT |
|  | Reverse | | GCCTCTGTAATCACTCATCACGA |
| *Srm* | Forward | | ACATCCTCGTCTTCCGCAGTA |
|  | Reverse | | GGCAGGTTGGCGATCATCT |
| *18s* | Forward | | CTCAACACGGGAAACCTCAC |
|  | Reverse | | CGCTCCACCAACTAAGAACG |

| **Table S3** LOD and LOQ of LC-MS/MS analysis | | | | |
| --- | --- | --- | --- | --- |
| Compounds | R^2^ | Linear (µg/mL) | LOQ (µg/mL) | LOD (µg/mL) |
| Cadaverine | 1 | 0.05 - 1 | 0.027 | 0.008 |
| Spermidine | 1 | 0.05 - 1 | 0.013 | 0.042 |
| Spermine | 0.996 | 0.05 - 1 | 0.176 | 0.053 |
| Ellagic acid | 0.980 | 1-50 | 2.31 | 0.69 |

**Supplementary Methods**

*Reagents and antibodies*

Ellagic acid (E2250), urolithin A (SML1791), urolithin B (SML1649), urolithin C (SML3047), urolithin D (SMB01339), CQ (C6628), CTB (C6499), dimethyl sulfoxide (317275), spermidine (S0266), spermine (85590), Baf (B1793), anti-vinculin antibody (V9131), anti-ULK1 antibody (A7481), and anti-p62 antibody (P0067) were purchased from Merck. X-tremeGEN 9 DNA transfection reagent (6365787001) and rapamycin (RM) (A-275) were purchased from Roche and ENZO Life Sciences, respectively. Torin 1 (4247) was purchased from Tocris Biosciences. Antibodies against p16 (sc-166760), p21 (sc-6246), p53 (sc-6243), α-tubulin (sc-5286), GAPDH (sc-365062), β-actin (sc-47778), MyoD1 (ac-32758), and Ac-lysine (sc-81623) were purchased from Santa Cruz Biotechnology. Antibodies against LC3B (2775), p-ULK1 (S757; 6888), p-ULK1 (S317; 37762), p-p62 (S403; 39786), EP300 (70088), p21 (2947), p-AMPK α (T172; 2535), AMPK α (2793), p-S6K1 (T389; 9205), S6K1 (2708), Ac-H3 (K56; 4243), and H3 (9715) were purchased from Cell signaling technology. Antibodies against Atg5 (NB110-53818) were purchased from Novus Biologicals. Murf1 (ab183094), Myf5 (ab69997), and FBXO32 (Atrogin-1; ab168372) were purchased from Abcam. Serum MCP1 (432704) and TNFα (432704) enzyme-linked immunosorbent assay kits were purchased from BioLegend.

*Autophagy activator screening using Cyto-ID*

Huh7 cells (60104, KCLB, Seoul, Korea) were cultured and incubated in high-glucose Dulbecco's Modified Eagle Medium (Hyclone, SH30243.01) supplemented with 10% fetal bovine serum (FBS, Hyclone, SH30919.03) and penicillin-streptomycin (Gibco, 15140122) at 37 ℃ with 5% CO2. For the assay, Huh7 cells were seeded into a 96-well black wall/clear-bottom plate, and then treated with each of 493 natural products in combination with CQ (10 μM). After 24 h, autophagy activation was assessed using the CYTO-ID® Autophagy detection kit (ENZ-51031, ENZO, NY, USA), following the manufacturer's instructions. Fluorescence intensity was measured using a fluorescein isothiocyanate filter (Ex 480/Em 530) and a 4',6-diamidino-2-phenylindole filter (Ex 340/Em 480) with an Infinite M200 microplate reader (Tecan US, Inc., NC, USA). The fluorescence intensity for each well was normalized against either CQ-treated cells or rapamycin + CQ-treated cells, and the results were displayed as a heatmap.

*Immunoblotting*

Proteins were extracted using cell lysis buffer (Cell Signaling Technology, 9803) and radioimmunoprecipitation assay buffer (Thermo Scientific, 89901) containing protease and phosphatase inhibitors (Thermo Scientific, 78440) for 20 min at 4 ℃. After centrifugation at 16,000 × g for 10 min at 4 ℃, the supernatant was harvested. The extracted proteins were quantified using the Pierce BCA Protein Assay (Thermo Scientific, 23227) and separated using sodium dodecyl-sulfate polyacrylamide gel electrophoresis. The proteins were transferred to a polyvinylidene fluoride membrane and blocked with 5% skim milk in tris-buffered saline with Tween for 1 h at room temperature. Next, the membrane was incubated with primary antibodies overnight at 4 ℃. The next day, the membranes were incubated with secondary antibodies dissolved in 5% skim milk for 1 h. Protein expression was detected using G:BOX Chemi XX6 (Syngene, MD, USA) with an ECL western substrate (Thermo Scientific, 32106).

*Immunofluorescence*

The mouse muscle tissue was embedded in an optimal cutting temperature compound and frozen. The samples were cut into 7-µm sections at -20 ℃ and fixed using 4% paraformaldehyde for 20 min. After blocking, the samples were stained with laminin antibody (1:200; Abcam, ab11575) at 4 ℃ overnight. After washing thrice, the samples were incubated with Alexa Fluor 488 goat anti-rabbit IgG (1:500; Cell Signaling Technology, 4412s) for 1 h at room temperature. Subsequently, the samples were mounted using Fluoroshield (Sigma-Aldrich, F6182) and captured using a confocal microscope (Olympus, FV3000), and the cross-sectional area was measured using ImageJ software.

*MTT assay*

C2C12 cells were seeded into a 96-well plate at a density of 1 × 10^4^ cells/well and incubated for 24 h. The cells were treated with various concentrations of *Castanea crenata* flower extract (CCFE). After 24 h, 20 μL of 3-(4,5-Dimethylthiazol-2-yl)-2,5-diphenyltetrazolium bromide (MTT solution, 5 mg/ml in phosphate-buffered saline) was added to wells and incubated for 4 h. All supernatants were removed, and 200 μL of dimethyl sulfoxide was added to the cells. The absorbance was measured at 540 nm using an Infinite M200 microplate reader (Tecan).

*C2C12 cell transfection and autophagy flux imaging*

C2C12 cells were seeded into a 4-well culture slide (SPL, 30114) and incubated for 24 h. The cells were transiently transfected with the mCherry-GFP-LC3 plasmid (250 ng/well) using X-tremeGENE™ 9 DNA transfection reagent (Roche, XTG9-RO), following the manufacturer’s instructions. After 24 h, the cells were washed with PBS, and fresh growth media were added. The cells were then treated with CCFE, bafilomycin A1, and rapamycin for 2 h. Fluorescence images were captured using a confocal microscope (Olympus FV3000), and both green and red dots were counted using ImageJ software (NIH, Bethesda, Maryland, USA) equibbed with the spot colocalization ComDet plugin (version 0.5.5).

*Human skeletal muscle cell line culture and cellular senescence induction*

Human skeletal muscle myoblasts (HSMMs) were purchased from Lonza (CC-2580) and cultured in SkBMTM-2 Basal Medium (Lonza, CC-3246) containing SkGMTM-2 SingleQuots ^TM^ supplements (Lonza, CC-3244). HSMMs were cultured until passage 12 (p12) to induce replicative cellular senescence. HSMMs (p7 and p12) were seeded into a 6-well plate and incubated. After 24 h, the cells were treated with CCFE (50–100 μg/ml) of rapamycin (RM) (50 nM) for 24 h and harvested for other experiments.

*Transmission electron microscopy imaging*

For TEM analysis, the gastrocnemius muscle, comprising both white and red muscle fibers, was fixed with 2% glutaraldehyde and 2% paraformaldehyde in 0.1 M PBS. Fixed skeletal muscles were washed with PBS and post-fixed with 1% osmium tetroxide (Electron Microscopy Sciences, 19152). After dehydration and infiltration, the muscles were embedded in a pure Epon 812 mixture. Samples were sectioned using an MT-X ultramicrotome (RMC, Tucson, AZ, USA) and collected on 100-mesh copper grids. After staining with 2% uranyl acetate and lead citrate, the samples were observed using Cryo-TEM (JEM-1400 Plus, 120 kV).

*mRNA sequencing*

Total RNA was isolated from mouse tibialis anterior muscle using Trizol reagent (Invitrogen). RNA quality was assessed with a TapeStation4000 System (Agilent Technologies, Amstelveen, The Netherlands), and RNA quantification was performed using an ND-2000 Spectrophotometer (Thermo Inc., DE, USA). Libraries were prepared from the total RNA using the CORALL RNA-Seq V2 Library Prep Kit (LEXOGEN, Inc., Austria). mRNA isolation was performed using the Poly(A) RNA Selection Kit (LEXOGEN, Inc., Austria), followed by cDNA synthesis and shearing following the manufacturer’s instructions. Indexing utilized Illumina indexes 1–12, and PCR was used for the enrichment step. Library quality was checked using the TapeStation HS D1000 Screen Tape (Agilent Technologies, Amstelveen, The Netherlands) to evaluate the mean fragment size. Quantification was performed using a library quantification kit with a StepOne Real-Time PCR System (Life Technologies, Inc., USA). High-throughput sequencing was performed as paired-end 100 sequencing on a NovaSeq 6000 (Illumina, Inc., USA). Quality control of the raw sequencing data was performed using FastQC [1]. Adapters and low-quality reads were removed using Fastp [2]. The trimmed reads were then mapped to the reference genome using STAR [3]. Read quantification was done using Salmon [4], and read counts were processed using the TMM + CPM normalization method via EdgeR [5]. Data mining and graphic visualization were performed using ExDEGA (Ebiogen Inc., Korea). Pathway enrichment analysis of differentially expressed genes (DEGs) was conducted using DAVID (https://david.ncifcrf.gov/ accessed on June 24, 2024). The Gene Ontology (GO) term enrichment analysis includes three attributes: molecular function (MF), biological process (BP), and cellular component (CC).

*UPLC-ESI-MS/MS analysis of mouse serum, liver, and kidney*

Mouse serum, liver, and kidney samples were acidified using 3 μL formic acid (pH 5), added with 8 μL of ß-glucuronidase from Helix pomatia, and incubated at 37 °C for 30 min at 120 rpm. The incubation was terminated using ice. Samples were extracted for 5 min using two volumes of ethyl acetate. After centrifugation at 3,000 x g for 5 min and phase separation, the upper organic solvent layer was evaporated and dried under nitrogen. The dry residues were dissolved with 200 μL of 50% methanol in an ultrasonic bath. Before LCMS/MS analysis, the extracted samples were centrifuged at 4 °C (15 min at 12,000 x g). The extraction samples (5 μL) were injected into a UPLC-MS/MS system (Acquity UPLC, Waters Corp., Milford, MA, USA) and separated using a BEH C18 column (2.1 x 100 mm, 1.7 μm, Waters) with a linear gradient of A (1% (v/v) formic acid in water) and B (1% (v/v) formic acid in acetonitrile). The composition was maintained at 5% B for 1 min, followed by a linear increase to 30% B by 4 min, a further increase to 90% B by 7 min, and then re-equilibration until 10 min. The column temperature and flow rate were 40 °C and 0.35 mL/min, respectively. The mass spectrometer was operated with an ESI interface in the positive ion mode. The MS parameter was optimized using a cone voltage of 28 V, a capillary voltage of 3.0 kV, and a collision energy of 21 V; the source temperature was set at 150 ℃, and the desolvation gas temperature was set at 500 ℃.

*HPLC analysis*

The dried extract was dissolved in 50% ethanol, sonicated for 20 min, centrifuged, and filtered using a 0.45-um syringe filter. The filtered sample was analyzed using high-performance liquid chromatography equipped with a YMC ODS-AM C18 (250 mm × 4.6 mm I.D. 5um) column. The mobile phase was 0.1% acetic acid (A) and 75% acetonitrile (B) containing 0.1% acetic acid, and the gradient was as follows: starting at 88% A, followed by 78% at 18 min, 62% at 35 min, 52% at 48 min, 32% at 54 min, 0% at 58 min, and 88% at 60 min. The flow rate was 1.0 mL/min, the sample injection volume was 10 uL, and the column temperature was 35 °C. Measurements were performed at wavelengths of 370 nm and 285 nm.

**References**

1. Simon A. FastQC. 2010. https://www.bioinformatics.babraham.ac.uk/projects/fastqc/.

2. Chen S, Zhou Y, Chen Y, Gu J. fastp: an ultra-fast all-in-one FASTQ preprocessor. Bioinformatics. 2018;34:i884-i90.

3. Dobin A, Davis CA, Schlesinger F, Drenkow J, Zaleski C, Jha S, et al. STAR: ultrafast universal RNA-seq aligner. Bioinformatics. 2013;29:15-21.

4. Patro R, Duggal G, Love MI, Irizarry RA, Kingsford C. Salmon provides fast and bias-aware quantification of transcript expression. Nat Methods. 2017;14:417-9.

5. Robinson MD, McCarthy DJ, Smyth GK. edgeR: a Bioconductor package for differential expression analysis of digital gene expression data. Bioinformatics. 2010;26:139-40.

**Supplementary references**

S1. Park S-H, Jeong HY, Choi PG, Kim MJ, Seo H-D, Hahm J-H, et al. C-Glycosidic flavone-rich *Passiflora incarnata* L. leaf extracts decrease body weight and fatty liver in obese mice. 2023;55:103028.

S2. Lee DH, Park SH, Huh YH, Jung Kim M, Seo HD, Ha TY, et al. Iridoids of *Valeriana fauriei* contribute to alleviating hepatic steatosis in obese mice by lipophagy. Biomed Pharmacother. 2020;125:109950.

S3. Katrukha, E. ekatrukha/ComDet: ComDet 0.5.5 (0.5.5). Zenodo. 2023.

S4. Kim YI, Lee H, Nirmala FS, Seo HD, Ha TY, Jung CH, et al. Antioxidant Activity of *Valeriana fauriei* Protects against Dexamethasone-Induced Muscle Atrophy. Oxid Med Cell Longev. 2022;2022:3645431.

S5. Lee H, Ha TY, Jung CH, Nirmala FS, Park SY, Huh YH, et al. Mitochondrial dysfunction in skeletal muscle contributes to the development of acute insulin resistance in mice. J Cachexia Sarcopenia Muscle. 2021;12:1925-39.

S6. Li R, Steyn FJ, Stout MB, Lee K, Cully TR, Calderon JC, et al. Development of a high-throughput method for real-time assessment of cellular metabolism in intact long skeletal muscle fibre bundles. J Physiol. 2016;594:7197-213.

S7. Matsumoto G, Wada K, Okuno M, Kurosawa M, Nukina N. Serine 403 phosphorylation of p62/SQSTM1 regulates selective autophagic clearance of ubiquitinated proteins. Mol Cell. 2011;44:279-89.

S8. Lopez A, Fleming A, Rubinsztein DC. Seeing is believing: methods to monitor vertebrate autophagy in vivo. Open Biol. 2018;8.

S9. Bang M, Kim DG, Gonzales EL, Kwon KJ, Shin CY. Etoposide Induces Mitochondrial Dysfunction and Cellular Senescence in Primary Cultured Rat Astrocytes. Biomol Ther (Seoul). 2019;27:530-9.

S10. Childs BG, Durik M, Baker DJ, van Deursen JM. Cellular senescence in aging and age-related disease: from mechanisms to therapy. Nat Med. 2015;21:1424-35.

S11. Kaushik S, Tasset I, Arias E, Pampliega O, Wong E, Martinez-Vicente M, et al. Autophagy and the hallmarks of aging. Ageing Res Rev. 2021;72:101468.
